# Dual Molecules Targeting 5-HT_6_ and GABA-A Receptors as a New Approach to Combat Depression Associated with Neuroinflammation

**DOI:** 10.1021/acschemneuro.3c00033

**Published:** 2023-04-04

**Authors:** Monika Marcinkowska, Barbara Mordyl, Agata Siwek, Monika Głuch-Lutwin, Tadeusz Karcz, Alicja Gawalska, Michał Sapa, Adam Bucki, Katarzyna Szafrańska, Bartosz Pomierny, Karolina Pytka, Magdalena Kotańska, Kamil Mika, Marcin Kolaczkowski

**Affiliations:** † Faculty of Pharmacy, Jagiellonian University Medical College, 9 Medyczna St., 30-688 Krakow, Poland; ‡ Adamed Pharma S.A., Pienkow, 6A Mariana Adamkiewicza St., 05-152 Czosnow, Poland

**Keywords:** GABA-A receptor, hybrid molecules, 5-HT_6_ receptor, anti-inflammatory activity, depression associated with inflammation, neuroinflammation

## Abstract

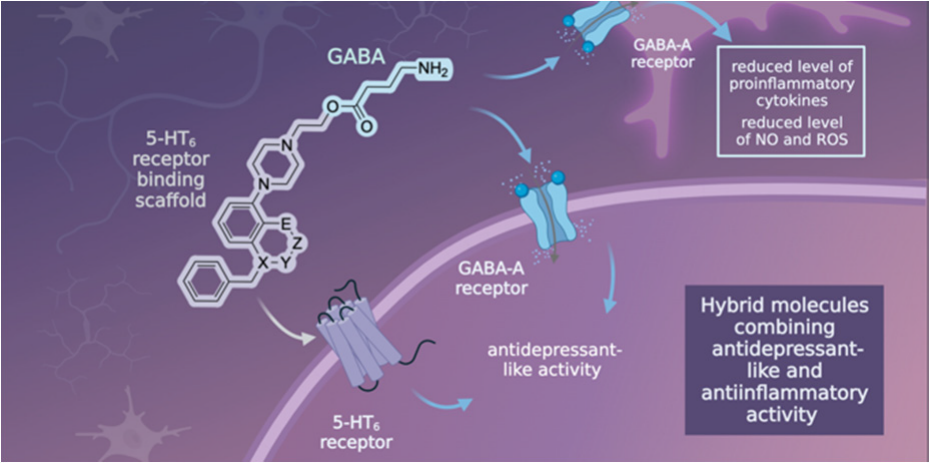

While monoaminergic deficits are evident in all depressed patients, nonresponders are characterized by impaired GABA-ergic signaling and the simultaneous presence of the inflammatory component. Pharmacological agents able to curb pathological immune responses and modulate ineffective GABA-ergic neurotransmission are thought to improve therapeutic outcomes in the treatment-resistant subgroup of depressed patients. Here, we report on a set of dually acting molecules designed to simultaneously modulate GABA-A and 5-HT_6_ receptor activity. The serotonin 5-HT_6_ receptor was chosen as a complementary molecular target, due to its promising antidepressant-like activities reported in animal studies. Within the study we identified that lead molecule **16** showed a desirable receptor profile and physicochemical properties. In pharmacological studies, **16** was able to reduce the secretion of proinflammatory cytokines and decrease oxidative stress markers. In animal studies, **16** exerted antidepressant-like activity deriving from a synergic interplay between 5-HT_6_ and GABA-A receptors. Altogether, the presented findings point to hybrid **16** as an interesting tool that interacts with pharmacologically relevant targets, matching the pathological dysfunction of depression associated with neuroinflammation.

## 1 Introduction

For decades, the primary objective of antidepressant therapy has been to increase the levels of monoamine neurotransmitters in the synaptic cleft.^1,2^ However, a selective focus on monoaminergic transmission is not effective for all patients. Despite the accessibility of the whole palette of antidepressants in the clinics, approximately 30% of patients do not respond to the marketed drugs.^3^ These data suggest that various mechanisms could play a role in the pathophysiology of depression, beyond monoamines. In this regard, several hypotheses were brought forward after clinical evidence confirmed changes in many neurotransmitter systems and various neurobiological variations.^4,5^

Significant progress has been made in understanding the specific neurochemical changes that play a role in the pathophysiology of treatment-resistant depression through brain imaging studies. For instance, magnetic resonance spectroscopy (MRS) revealed a significant reduction in GABA levels in plasma, cerebrospinal fluid, and cortical regions of depressed subjects.^6^ Neurochemical changes are consistent with changes in the levels and activity of GABA-A receptors.^7^ These findings, along with other neurochemical evidence, gave rise to the “GABA-ergic hypothesis of depression”.^5^ Highly noteworthy, GABA deficits are particularly evident in the subgroup of treatment-resistant patients. ^1^H MRS brain scans revealed substantially lower levels of GABA in the frontal cortex in the treatment resistant subgroup compared to patients without a history of treatment resistance.^8^ These findings suggest that compounds modulating the GABA-ergic signaling may help to optimize the patient’s response by interacting with the relevant target for the disease.

A plethora of evidence has confirmed that neuroinflammation is a widely recognized component of the pathophysiology of depression.^4,9,10^ Clinical studies showed that depressed patients suffer from increased serum levels of proinflammatory cytokines, particularly interleukin IL-6, IL-1β, and tumor necrosis α factor (TNF-α).^9,11^ Several clinical studies observed a robust association between a raised level of IL-6 and anhedonic states.^12^ Further studies indicated that persistent neuroinflammation may sabotage a patient’s response to the marketed antidepressants.^13,14^ The TNF-α was found to enhance the activity of neuronal SERT via stimulation of p38 MAPK.^15^ Therefore, the coexisting inflammatory factors circumvent the primary mechanism of serotonergic medications and can contribute to treatment resistance. Consequently, this scenario may be present in nearly 30% of all depressed subjects.^3^

Given the disappointing state related to the current pharmacotherapy for depression, the development of small molecule therapeutics with a novel mechanism of action has been intensively pursued to optimize the patient’s response to treatment. Interestingly it is possible to simultaneously target inflammatory responses and impaired GABA-ergic signaling with molecules modulating the activity of GABA-A receptors.^16^ Both microglia and neurons express GABA-A receptors, and modulation of microglia activity via GABA-A receptors decreases secreting proinflammatory cytokines IL-6 and TNF-α.^16−18^ At the same time, modulation of neuronal GABA-A receptor activity might regulate the impaired GABA-signaling.^7^

**Figure 1:**
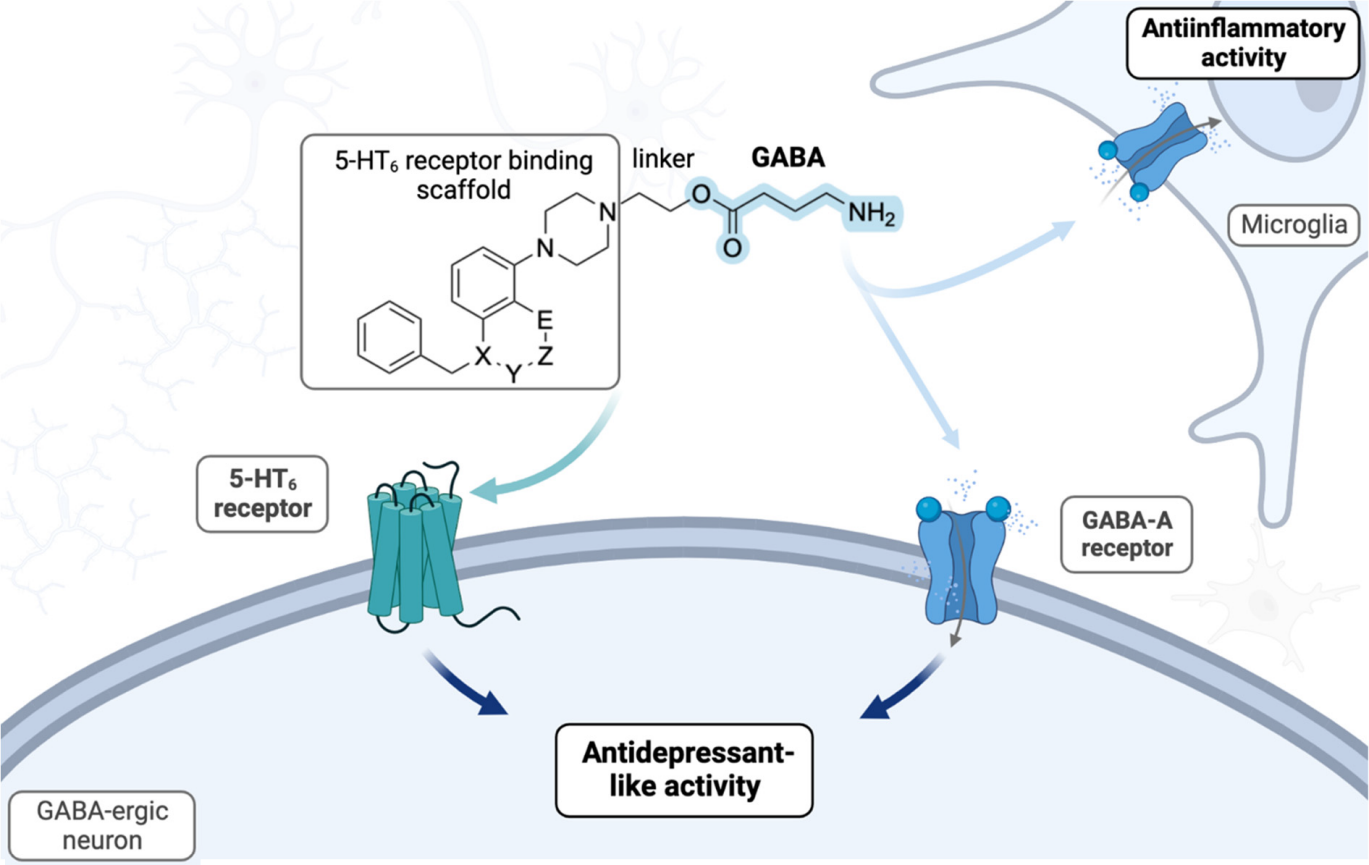
General concept of hybrid molecules targeting GABA-A and 5-HT_6_ receptors, employing novel 5-HT_6_ binding chemotypes (5-HT_6_ antagonism): X = N, O; Y = CO, CH_2_, none; Z = CH_2_, none; E = N, O, CH_3_.

Inspired by the pallet of functions that GABA-A receptors can offer to mitigate depression, we designed a set of dually acting compounds that harness the GABA molecule, which is presumed to exert anti-inflammatory activity and antidepressant properties (Figure 1). The GABA molecule was assembled with a chemical scaffold that interacts with a complementary biological target, involved in the regulation of mood deficits, namely, the serotonin 5-HT_6_ receptor. Considering that regions of the brain involved in regulating emotions and memory processes (cortex and hippocampus) express the 5-HT_6_ receptor, we reasoned that it can be beneficial for therapeutic purposes.^19^ Both 5-HT_6_ agonists and 5-HT_6_ antagonists have demonstrated antidepressant-like activity in animal models. However, the current landscape of small molecules acting as 5-HT_6_ antagonists appears to be more developed.^20^ In fact, selective 5-HT_6_ antagonists hold great promise as small molecule therapeutics in neuropsychiatric diseases, due to their promising antidepressant-like efficacy in animal models.^21^ Continuing our previous research in this area,^22,23^ in the present work, we explored the novel 5-HT_6_ binding chemotypes bearing various heterocycles, which could be easily connected with the function of GABA to construct a series of bifunctional molecules **16**–**20**. Within the *in vitro* profiling cascade, we identified hybrid molecule **16** characterized by the most desirable receptor profile and drug-like properties. Compound **16** was then assayed in BV-2 microglia cells to explore its ability to attenuate neuroinflammation. To verify the therapeutic potential of this novel chemotype, **16** was subsequently characterized in *in vivo* studies.

## 2 Results and Discussion

### 2.1 Design and Synthesis

**Scheme 1:**
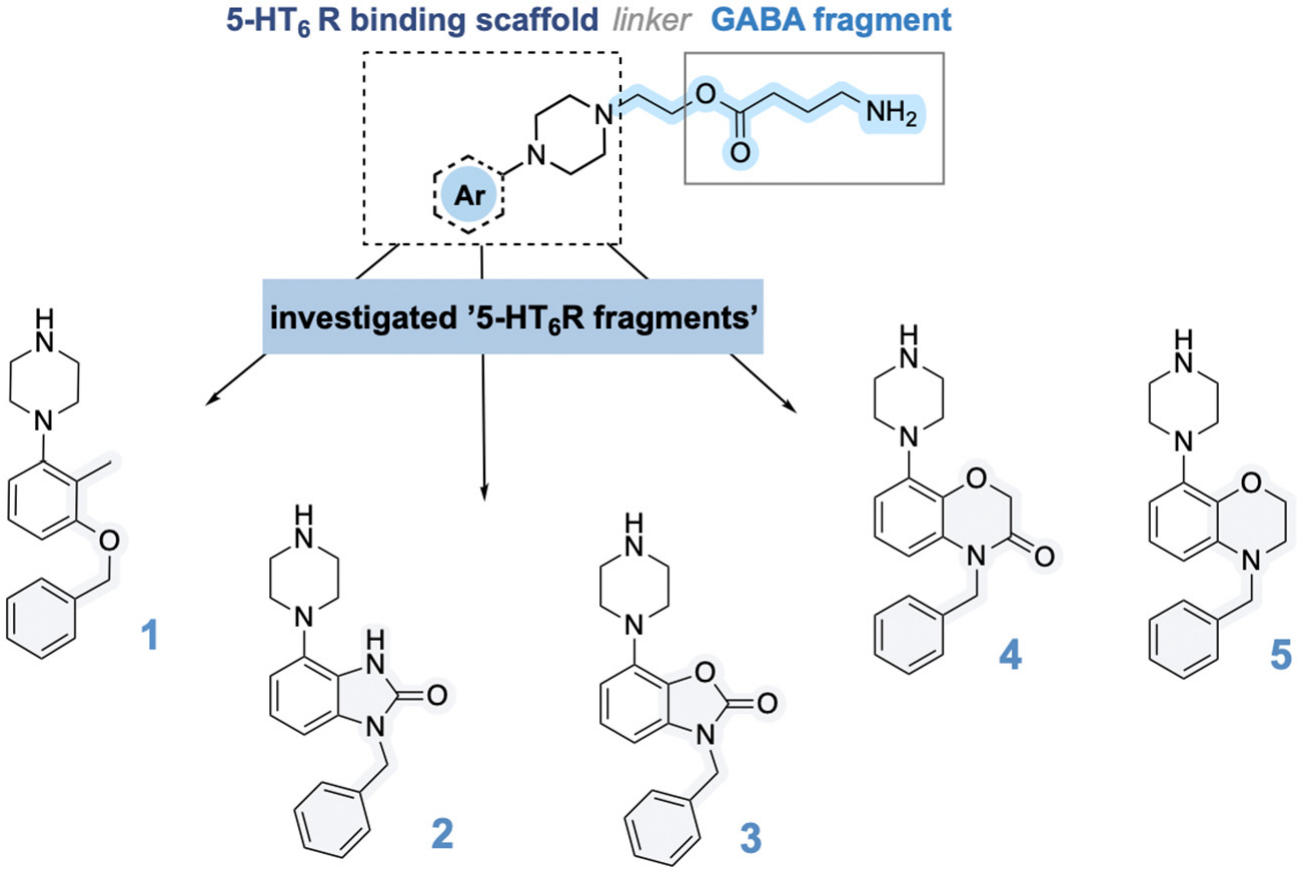
Design Concept and Chemical Structure of a Series of Bifunctional Molecules The shaded portion of the molecule illustrates the key variations between the 5-HT_6_ binding scaffolds used.

Previously we have found that anchoring the GABA molecule via ethyl ester linker with a 5-HT_6_ receptor blocking scaffold yields dually active molecules, characterized by favorable chemical stability and optimal brain penetration.^22^ This strategy was confirmed for a series of indole derivatives acting as a 5-HT_6_ receptor binding scaffolds. The compounds containing the sulfonamide group showed superior activity, apparently due to hydrogen bonding with the Asn6.55 side chain. In the pursuit of novel biologically active chemotypes, in the present work we replaced the indole rings with the 5-HT_6_ receptor binding scaffolds containing hydrogen bond acceptors that would secure both the favorable interactions and physicochemical properties: 3-(benzyloxy)-2-methylphenyl (**1**),^24^ 1,3-dihydro-2*H*-benzo[*d*]imidazol-2-one (**2**),^25^ and 2*H*-benzo[*b*][1,4]oxazin-3(4*H*)-one (**4**).^26^ Based on the molecular modeling studies described in detail in the next section, we presumed that the novel scaffolds would be beneficial for the interactions with the 5-HT_6_ receptor, due to the carbonyl/ether group that would allow for more efficient interaction with Asn6.55 which in turn stabilizes the aromatic interaction with Phe5.38. Alongside, we decided to investigate novel 5-HT_6_ binding fragments: 3-benzyl-7-(piperazin-1-yl)benzo[*d*]oxazol-2(3*H*)-one (**3**) and 4-benzyl-8-(piperazin-1-yl)-3,4-dihydro-2*H*-benzo[*b*][1,4]oxazine (**5**), which represent bioisosters of **2** and **4**. Therefore, all the above-mentioned 5-HT_6_ receptor antagonism fragments were incorporated with GABA ethoxy moiety to compose a set of GABA/5-HT_6_ receptor hybrids (Scheme 1).

**Scheme 2:**
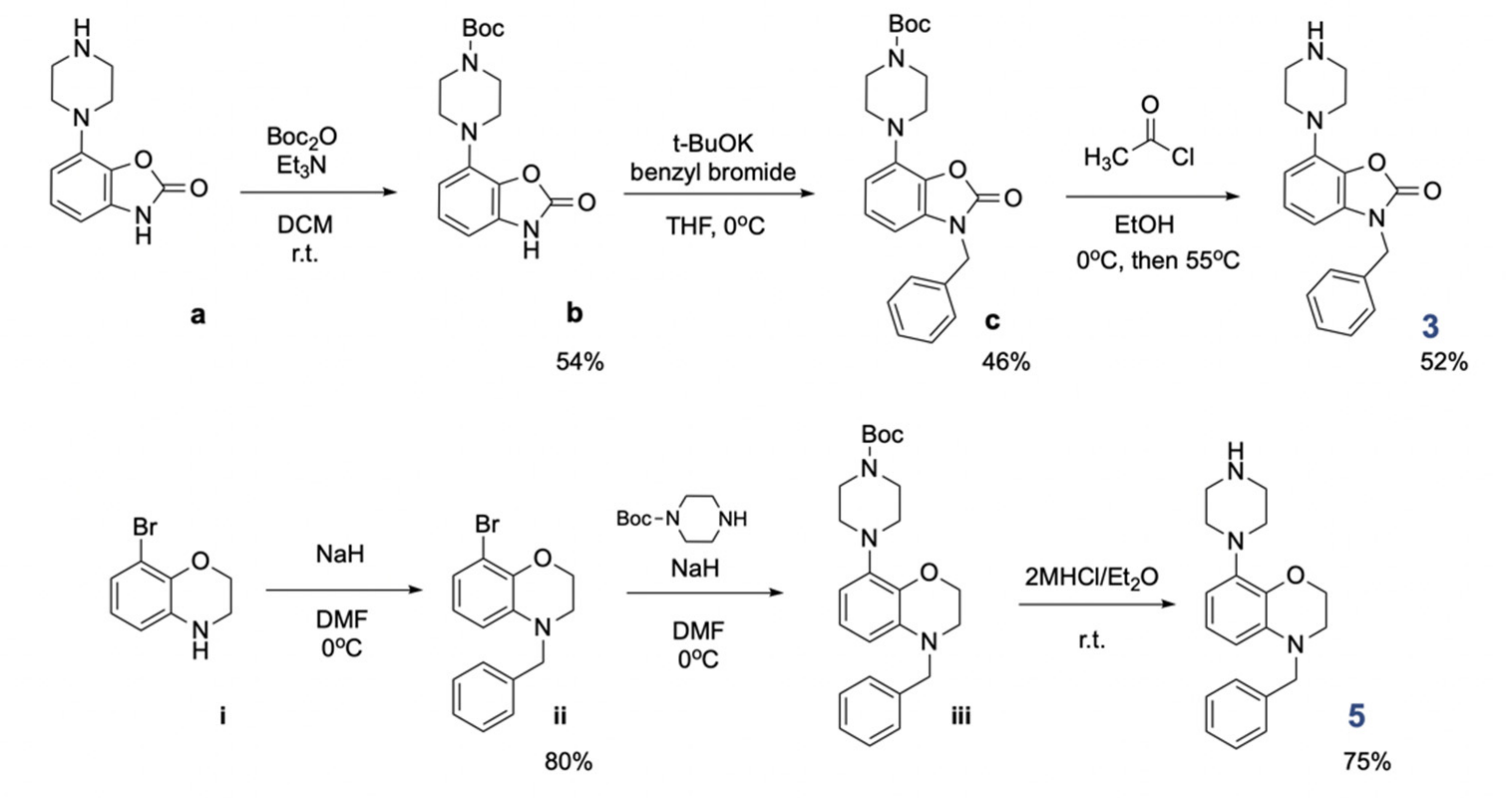
Preparation of Key Building Blocks **3** and **5**

**Scheme 3:**
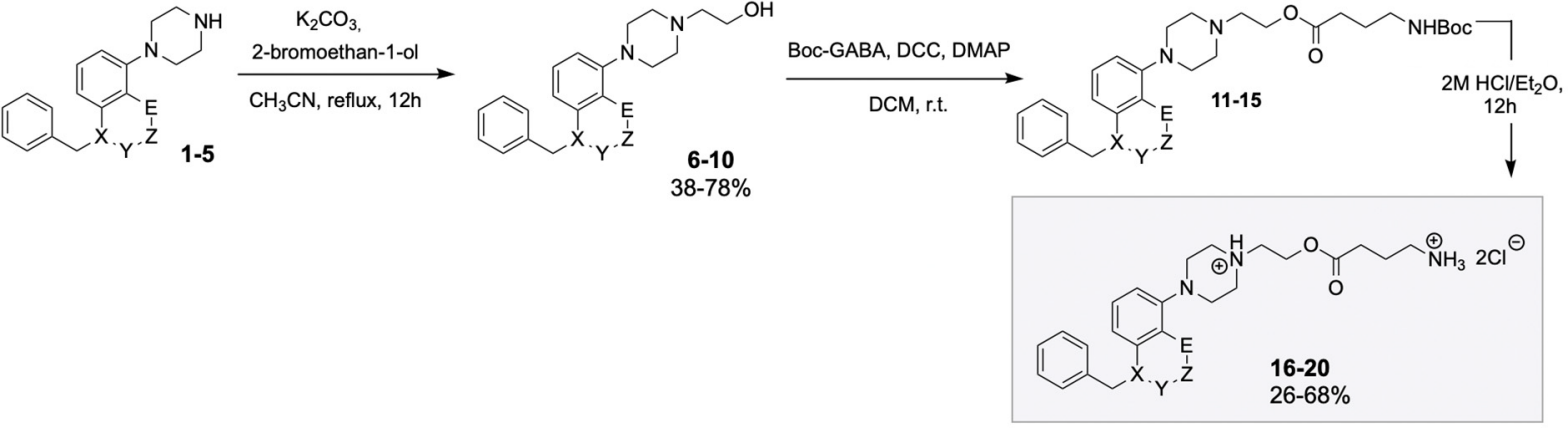
Synthesis of Final Hybrid Molecules **16**–**20** X = N, O; Y = CO, CH_2_, none; Z = CH_2_, none; E = N, O, CH_3_.

The key 5-HT_6_ antagonists (**1**,^24^**2**,^25^ and **4**(26)) were synthesized according to previous protocols, while the **3** and **5** building blocks were prepared in our laboratory according to Scheme 2. Starting from the synthesis of 5-HT_6_ receptor binding scaffold **3**, Boc protection of 7-(piperazin-1-yl)benzo[*d*]oxazol-2(3*H*)-one (**a**) delivered intermediate **b**, followed by functionalization with benzyl bromide to give **c** precursor. The key building block **3** was delivered by gentle deprotection using acetyl chloride in EtOH. The 5-HT_6_ scaffold **5** was prepared in three-step synthesis, starting from installation of benzyl moiety to deliver **ii** derivative. The latter was reacted with Boc-piperazine ring to deliver **iii** intermediate. Removal of Boc protecting moiety using 2 M HCl/Et_2_O liberated the final building block **5**. With both building blocks **3** and **5** in hand, we proceeded with the synthesis of hybrid molecules **16**–**20** (Scheme 3). The key building blocks **1**–**5** were functionalized with bromoethanol to deliver **6**–**10** alcohols. Next, the alcohols **6**–**10** were reacted with Boc-GABA to deliver ester intermediates **11**–**15**. Ultimate deprotection of **11**–**15** esters with HCl/Et_2_O liberated final molecules **16**–**20** in the form of dichloride salts (^1^H NMR spectra available in the Supporting Information).

### 2.2 Structure–Activity Relationships

*In vitro* radioligand binding studies were conducted to evaluate the affinity of all hybrid molecules for the muscimol site of the GABA-A receptor and the serotonin 5-HT_6_ receptor. Our starting point focused on determination of the affinity of **1**–**5** scaffolds for the 5-HT_6_ receptor. Consistent with previous reports, **1**, **2**, and **4** exerted nanomolar affinity for the 5-HT_6_ receptor (*K*_i_ = 0.2–2.7 nM). The novel fragments **3** and **5** also showed a high affinity with *K*_i_ values of 4.0 ± 0.2 nM and 60.0 ± 7.5 nM, respectively (Table 1). Next, we focused our attention on assessing the impact of the incorporation of the alkyl linker into **1**–**5** scaffolds. We observed that alcohols **6**, **9**, and **10** performed slightly better and maintained a high affinity for the 5-HT_6_ receptor (Table 1). The installation of the ethoxy chain in **6**, **9**, and **10** induced a minimal alteration in the affinity for the receptor of interest. However, the introduction of the ethoxy chain to the 3-benzyl-7-(piperazin-1-yl)benzo[*d*]oxazol-2(3*H*)-one scaffold (compound **8**) and to the 1-benzyl-4-(piperazin-1-yl)-1,3-dihydro-2*H*-benzo[*d*]imidazol-2-one ring (**7**) led to a slight decrease in affinity for the 5-HT_6_ receptor (**7**, *K*_i_ = 40.0 ± 3.0 nM; **8**, *K*_i_ = 150.0 ± 7.6 nM). The installation of the GABA function in the final hybrid molecules did not change the affinity for the 5-HT_6_ receptor. This conclusion is true for **16**, **17**, **18**, and **19** hybrids. Hybrid molecules bearing 1-(3-(benzyloxy)-2-methylphenyl)piperazine (**16**) and 4-benzyl-8-(piperazin-1-yl)-2*H*-benzo[*b*][1,4]oxazin-3(4*H*)-one ring (**19**) showed the highest potency (**16**, *K*_i_ = 16.0 ± 0.4 nM; **19**, *K*_i_ = 25.0 ± 1.0 nM) among all the series. Solely the 4-benzyl-8-(piperazin-1-yl)-3,4-dihydro-2*H*-benzo[*b*][1,4]oxazine analogue (**20**) did not show desired affinity (*K*_i_ = 1260.0 ± 82.0 nM). Then, the compounds **16**–**20** were subjected to functional studies, which showed that the compounds exhibited notable antagonist effectiveness at the 5-HT_6_ receptor, with *K*_B_ values ranging from 11.9 ± 0.1 to 1670 ± 1.5 nM. The most effective antagonistic responses were observed for compounds **19** and **16** with *K*_B_ values of 7 ± 0.1 and 11.9 ± 0.1.

**Table 1:**
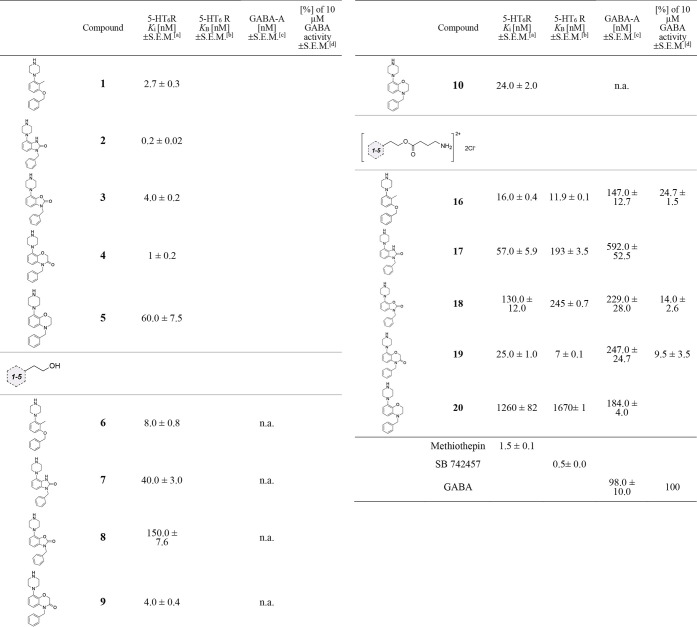
Radioligand Binding Affinity of **1**–**5**, **6**–**10**, and **16**–**20** for 5-HT_6_ Receptor and the Muscimol Site of the GABA-A Receptor: Antagonistic Activity at 5-HT_6_R (*K*_B_) and Electrophysiological Studies of the Selected Compounds

a The values of affinity are expressed as *K*_i_ and ± SEM and were determined from minimum three experiments conducted in duplicate. Competitive replacement of [^3^H]-LSD was used to measure the affinities for the 5-HT_6_ receptor.

b The outcomes were standardized as a percentage of the maximum reaction observed in the absence of the antagonist.

c Competitive replacement of [^3^H]muscimol was used to establish the affinities for the GABA-A receptor.

d The electrophysiological studies examined the agonist mode of tested compounds at a concentration of 10 μM. Results are represented by the GABA current amplitude normalized to 10 μM GABA-induced response (10 μM). n.a.: “not active”. Blank spaces: not tested.

We next proceeded with the investigation of the affinity for the GABA-A receptor (Table 1). Considering that the GABA neurotransmitter binds to the GABA-A receptor at the muscimol site, we chose this site in the radioligand binding assay as it closely mimics the physiological activity of the receptor.^27^ Hybrids **16** and **20** displayed a binding affinity (*K*_i_ = 147.0 ± 12.7 nM, *K*_i_ = 184.0 ± 4.0 nM) close to the natural GABA neurotransmitter (*K*_i_ = 98 nM). The measured affinities for the remaining GABA derivatives (**18** and **19**) were *K*_i_ = 229.0 ± 28.0 nM for **18** and *K*_i_ = 247.0 ± 24.7 nM for **19**. In the case of 1-benzyl-4-(piperazin-1-yl)-1,3-dihydro-2*H*-benzo[*d*]imidazol-2-one derivative **17** the affinity for GABA-A receptor dropped to 592.0 ± 52.5 nM. To confirm that the compounds interact with the receptors as hybrids and not hydrolysis products, we tested the stability of a representative hybrid **16** under radioligand binding assay conditions and found 98.21% of the compound remaining, after 1 h of incubation with brain tissue.

Based on the overall results of the radioligand binding assays, for electrophysiological studies we selected the most promising hybrids (**16**, **18**, and **19**), considering the complexity of the studies. The selection criteria focused on molecules that had an affinity for both the GABA-A and 5-HT_6_ receptors, with a *K*_i_ value of less than 250 nM. Therefore, molecules **16**, **18**, and **19** satisfied these criteria and were further evaluated in functional studies. The effects of selected hybrids molecules were evaluated at 10 μM concentration using a HEK293 cell line stably expressing human α_1_β_2_γ_2_-GABA-A receptors and were compared with effects provoked by GABA alone (at 10 μM concentration). According to the protocol, the intensity of the electrical current evoked by GABA was measured and established as the baseline value of 100%. The amplitudes of the compounds tested were then measured and expressed as a percentage of the amplitude produced by the natural GABA agonist (Table 1). Considering that GABA acts as an agonist at the GABA-A receptor, we hypothesized that the hybrid molecules containing a GABA ester moiety would also act similarly.^28^ We observed that the tested compounds induced an increase in the ion current, displaying weak agonistic properties. The increase in ion current for **16**, **18**, and **19** was 24.7% ± 1.5, 14.0% ± 2.6, and 9.5% ± 3.5 of the GABA responses (Table 1).

### 2.3 Molecular Modeling Studies

Data from radioligand binding studies were supported with *in silico* studies to survey the contribution of molecular interactions between both molecular targets: GABA-A and 5-HT_6_ receptors and representative hybrid molecule **16**. Based on the overall receptor profile, we selected compound **16** as the main structure for further investigation, which showed a balanced receptor profile (*K*_i_ < 150 nM for both targets).

**Figure 2:**
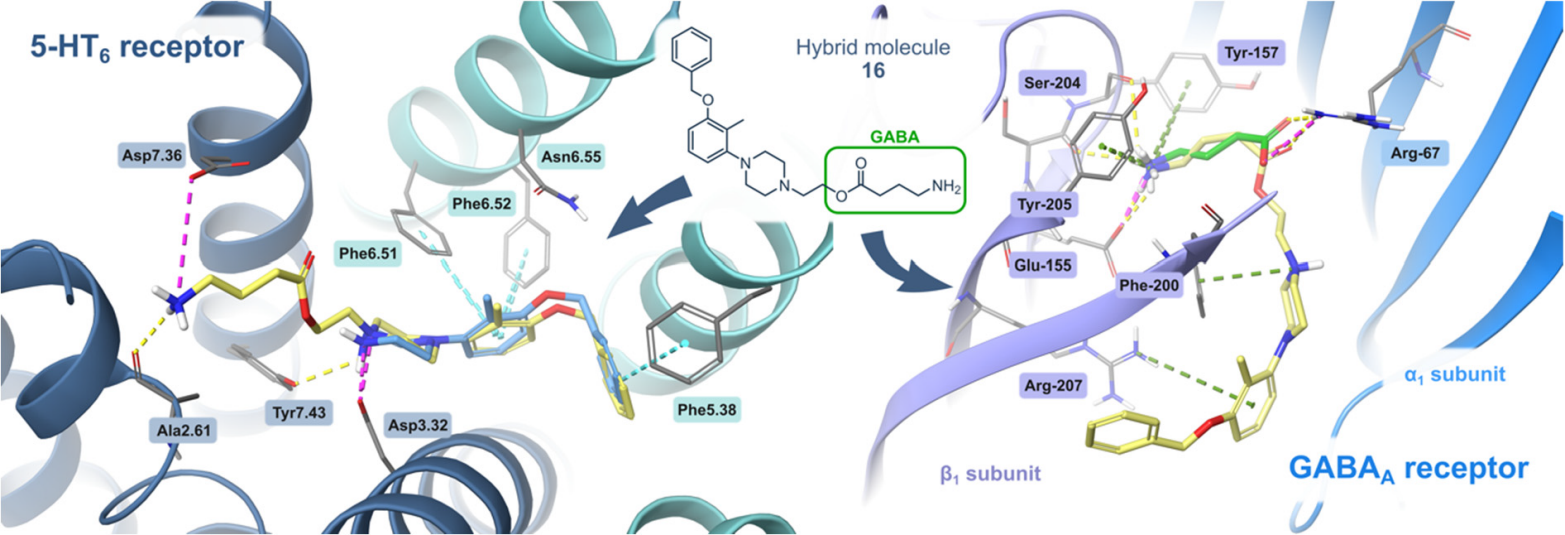
Potential binding mode of **16** at the orthostatic sites of both targets: left, the 5-HT_6_ receptor; right, GABA-A receptor (canary, **16**; cyan, 5-HT_6_ receptor ligand **1**; green, GABA). 1-[3-(Benzyloxy)-2-methylphenyl]piperazine moiety of **16** established equal interactions to selective 5-HT_6_ antagonist **1**, inside the 5-HT_6_ receptor. In the GABA-A receptor, the acyl fragment of **16** exhibited a binding pose akin to that of the natural agonist GABA. The remaining hybrid elements did not induce steric hindrance in the active sites of 5-HT_6_ and GABA-A receptors.

The predicted binding mode of a representative hybrid molecule **16** was similar to that observed for the selective 5-HT_6_ antagonist (**1**), and both molecules were anchored at the primary binding site, inside the 5-HT_6_ receptor (Figure 2). We observed that 1-(3-(benzyloxy)-2-methylphenyl)piperazine scaffold formed a salt bridge between Asp3.32 and the protonated nitrogen atom of **16**, stabilized by π–π stackings with Phe6.51/6.52, the characteristic interactions observed for GPCRs. The benzyl moiety of hybrid **16** occupied a hydrophobic pocket between Val3.33 and Ala5.42 residues, interacting with Phe5.38. The analysis of the interactions of the compound series resulting from the scaffold hopping suggested the importance of a hydrogen bond acceptor exposed in the region of the polar interactions with Asn6.55. The superior affinity of compound **19** may be explained by the privileged formation of an H-bond with the latter residue (see Supporting Information), and its importance was confirmed by the drop in the affinity of compound **20**. The ethyl linker and GABA fragment of **16** were anchored in an external pocket between TMH1, TMH2, and TMH7, and the protonated amine group of GABA formed ionic interaction with Asp7.36. This GABA fragment extended beyond the orthosteric binding site and did not cause a steric hindrance.

Further studies suggested that the **16** interacts with an orthosteric binding site of the α_1_β_3_γ_2_ GABA-A receptor, which is situated within the α_1_ and β_3_ subunits (Figure 2). Analogous to the natural agonist GABA, the GABA-fragment of **16** adopted an identical position in the cryo-EM structure and produced equivalent interactions. We could observe hydrogen bonds with both α_1_Arg-67 and β_3_Ser-156, a cation−π aromatic interaction with β_3_Tyr-205, and an ionic bond formed with β_3_Glu-155. The binding pose was also stabilized by the cation−π interactions formed between β_3_Phe-200 and protonated piperazine moiety, as well as β_3_Arg-207 and the benzyl ring, respectively. The remaining derivatives were characterized by an analogous binding mode, with the difference that in the case of hybrids **17** and **18** there was a lack of cation−π interaction with β_3_Arg-207 (corresponding figures in the Supporting Information).

### 2.4 Physicochemical and Preliminary ADMET Profiling

In addition to potency, key features that govern successful therapeutic-like activity are delineated by physicochemical profile. Selected hybrid **16** was subjected to further *in vitro* physicochemical profiling to determine its chemical stability in pH = 7.4, metabolic stability, solubility, and passive permeability (Table 2). In the thermodynamic solubility assay (PBS pH = 7.4), compound **16** was characterized by high aqueous solubility of >2 mg/mL. The parallel artificial membrane permeability assay (PAMPA) revealed that **16** exerts satisfying permeability (6.24 × 10^–6^ cm/s), suggesting good penetration through biological barriers. The metabolic stability evaluated using RLMs (rat liver microsomes) indicated a high metabolic stability of **16** (Table 2). Hepatotoxicity studies using HepG2 cells viability assay and highly sensitive probe ToxiLight^29^ indicated a not significant reduction of viability in concentrations up to 50 μM (see the Supporting Information). In addition, we tested selected compound **16** in various stability assays, including chemical stability in PBS, rat plasma stability, and brain tissue stability. Compound **16** displayed a desirable stability level ranging from 79.65 to 92.46% after 60 min under different conditions (Table 3). Overall, based on the results presented, we expected that **16** may produce the desired level of *in vivo* efficacy.

**Table 2:** Selected *in Vitro* Assays for **16** and the Reference Molecules

compound	solubility (pH = 7.4, mg/mL)^a^	PAMPA permeability (10^–6^ cm/s)^b^	rat liver microsomes *t*_0,5_ [min]^c^	rat liver microsomes Cl_int_ ((μL/min)/mg)^c^
**16**	>2	6.24	>60	<115.5
perphenazine	0.0306			
sulpiride		0.016		
propranolol			>60	<115.5

a Thermodynamic solubility measured at rt.

b Compounds tested at 10^–5^ M using the PAMPA Plate System Gentest.

c Molecule assayed at 10^–7^ M. Empty cells: not examined.

**Table 3:** Stability Assays for **16** (Chemical Stability in PBS, Plasma, and Brain Homogenate)

time point [min]	% compound remaining, rt^a^	% compound remaining (rat plasma, 37 °C)	% compound remaining (brain homogenate)^b^
0	100 ± 0.2	100 ± 0.1	100 ± 0.3
15	98.43 ± 1.8	97.21 ± 0.8	97.81 ± 2.1
30	94.23 ± 1.8	90.35 ± 1.4	92.56 ± 1.9
60	92.46 ± 2.2	79.65 ± 2.5	87.12 ± 3.2
120	86.63 ± 2.3	45.37 ± 2.2	49.73 ± 2.4
240	62.21 ± 1.7	21.46 ± 1.2	25.34 ± 1.6

a Assay performed in PBS, rt.

b Compound **16** was incubated with brain homogenate suspended in Tris-HCl buffer.

### 2.5 *In Vitro* Evaluation of Anti-Inflammatory Properties of **16**

**Figure 3:**
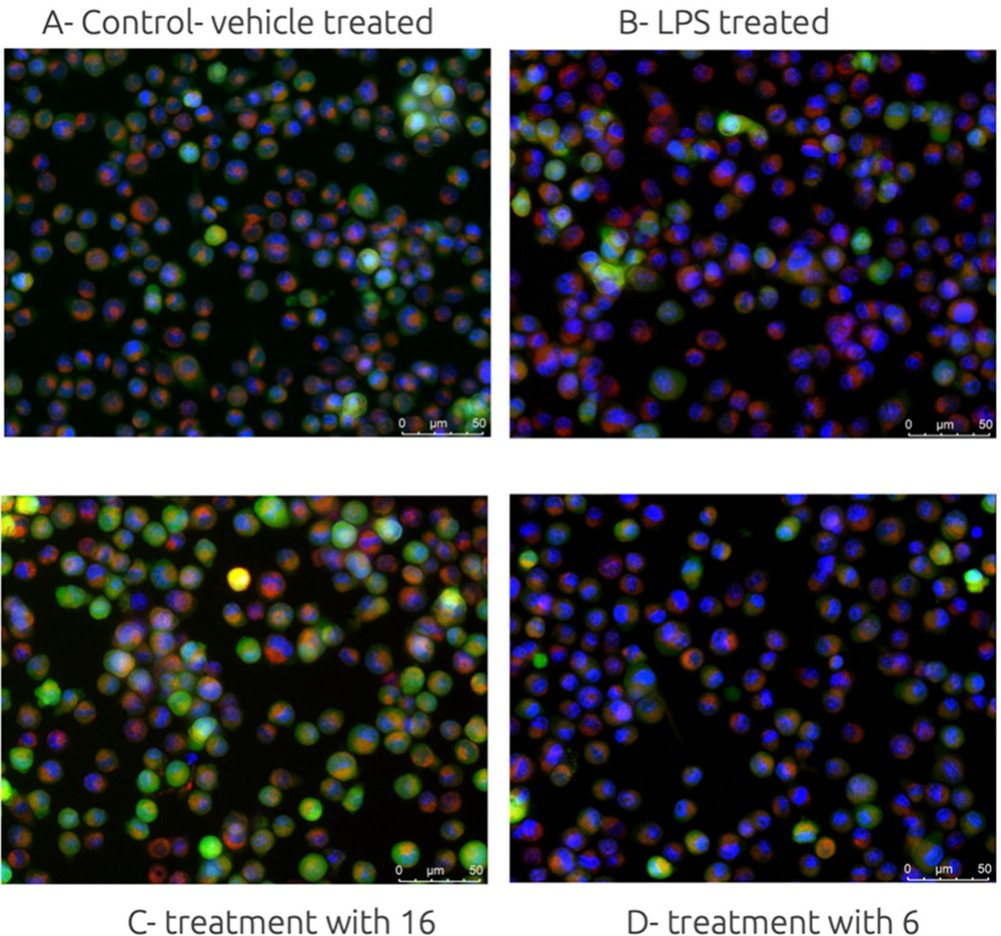
Representative images of microglia BV-2 cells: (A) control, vehicle treated cells, 0.1% DMSO; (B) cells treated with LPS (18 h); (C) cell pretreated with hybrid molecule **16** (10 μM, 1h); (D) cell pretreatment with alcohol **6** (10 μM, 1 h). BV-2 cells were stained with Calcein AM to highlight the outer membranes (green), Hoechst 33342 to detect the nucleus (blue), and MitoTracker to stain active mitochondria (red).

Activated microglia may disrupt neurotransmitter balance via enhanced production of proinflammatory cytokines and secretion of oxidative stress markers.^11,10^ Considering that modulation of the microglia’s activity by small molecules can be harnessed for therapeutic purposes, we sought to evaluate the potential of **16** to control inflammatory reactions. The relevance of this approach has been fueled by the fact that inflammatory signaling from microglia might be modulated by GABA-A receptor ligands.^30,31^ To stimulate an inflammatory response and oxidative stress, the microglia BV-2 cells were subjected to treatment with lipopolysaccharide (LPS) (Figure 3). The LPS stimulation assay reflects the clinical settings in which patients exposed to immune activation by bacterial LPS were characterized by depression symptoms.^32^ In first-line experiments, we assessed the morphology of microglial cells exposed to LPS and pretreated with selected ligands using fluorescence microscopy. Upon stimulation with LPS, we observed that mitochondrial metabolism and respiration in BV-2 cells changed, as there was a clear change in mitochondrial membrane potential (Figure 3B), detected with a fluorescent dye MitoTracker.^33^ The observed red fluorescence denoted elevated activation of mitochondria after stimulation with LPS (Figure 3B). In contrast, pretreatment with **16** (10 μM), protected the microglia from LPS insult, given that the red fluorescence was negligible and the fluorescence we observed was predominantly blue and green, characteristic for the cytoplasm and nucleus. When cells were pretreated with alcohol **6** (5-HT_6_ antagonist part deprived of the GABA part), we observed that the mitochondrial membrane potential was changed, suggesting that the alcohol **6** was deprived anti-inflammatory activity and the observed anti-inflammatory effects of **16** was arising from the modulation of GABA-A receptor activity.

**Figure 4:**
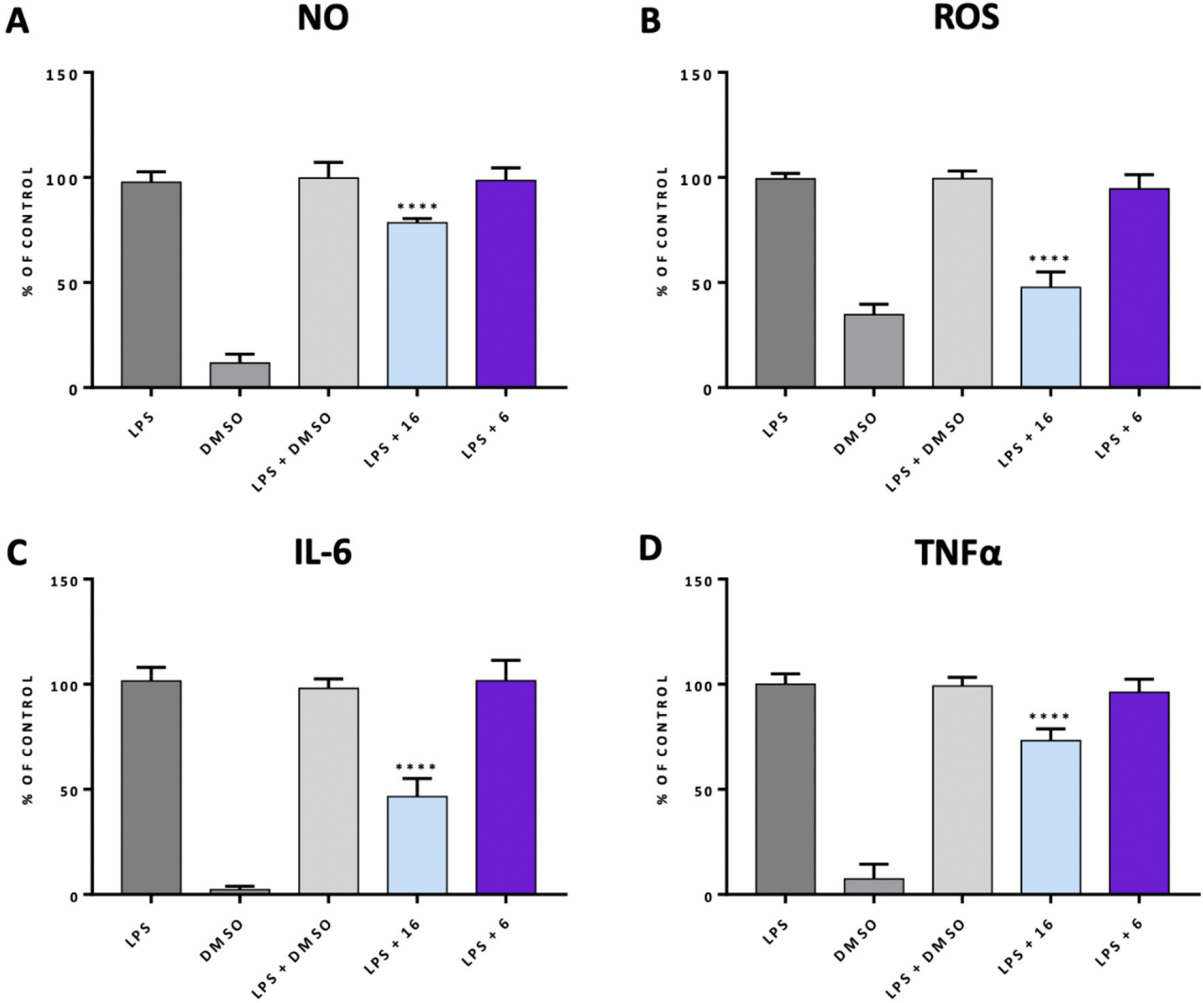
Determination of anti-inflammatory activity of **16** in comparison to the activity of **6**. Pretreatment with **16** or **6** (10 μM), DMSO (0.1%, v/v) for 1 h followed by incubation with LPS for 18 h and measurement of nitric oxide (A), reactive oxygen species (B), IL-6 (C), and TNF-α (D). The results are reported as a percentage of the maximum cellular response to the LPS stimulation. The reported values are represented as the mean ± SD. To determine the differences between the treatment groups and the LPS-treated group, a one-way ANOVA was used, followed by post hoc analysis (Dunnett’s multiple comparison tests). Statistical significance was established if *p* < 0.05 (****p < 0.0001). The statistical analysis was conducted utilizing the GraphPad software. The studies were conducted using a plate reader (POLARstar Omega, BMG).

In the second line of experiments we aimed to establish if the hybrid molecule **16** can abolish oxidative stress and decrease the level of inflammatory markers. In response to LPS stimulation, microglia produced a substantial amount of toxic insults such as NO and ROS and proinflammatory cytokines IL-6 and TNF-α compared to the control group (Figure 4). In the group where BV-2 cells were pretreated with alcohol **6**, the levels of ROS, NO, and proinflammatory mediators (IL-6 and TNF-α) did not change. On the contrary, pretreatment with **16** significantly decreased LPS-induced ROS, NO, IL-6, and TNF-α production, indicating anti-inflammatory activity of **16** (Figure 4). These results are in line with our previous studies in which we reported the anti-inflammatory efficacy of the GABA-A/5-HT_6_ hybrid molecules.^22^

### 2.6 *In Vivo* Behavioral Studies

To test the *in vivo* antidepressant-like efficacy of selected lead molecule **16**, we employed a forced swim test (FST, Porsolt test), due to its high predictive validity for selecting a broad range of antidepressant agents.^34^ The behavioral studies were carried out using female rats, given that the prevalence of depression in female subjects is estimated to double compared to male. Moreover, female subjects are particularly prone to fluctuations in GABA-ergic system.^35^ In the given experimental model, the activity of hybrid **16** was compared to the efficacy of parent alcohol **6** (5-HT_6_ antagonist part deprived of GABA function). GABA was not tested, as it does not produce antidepressant effects, as we reported previously.^22^

**Figure 5:**
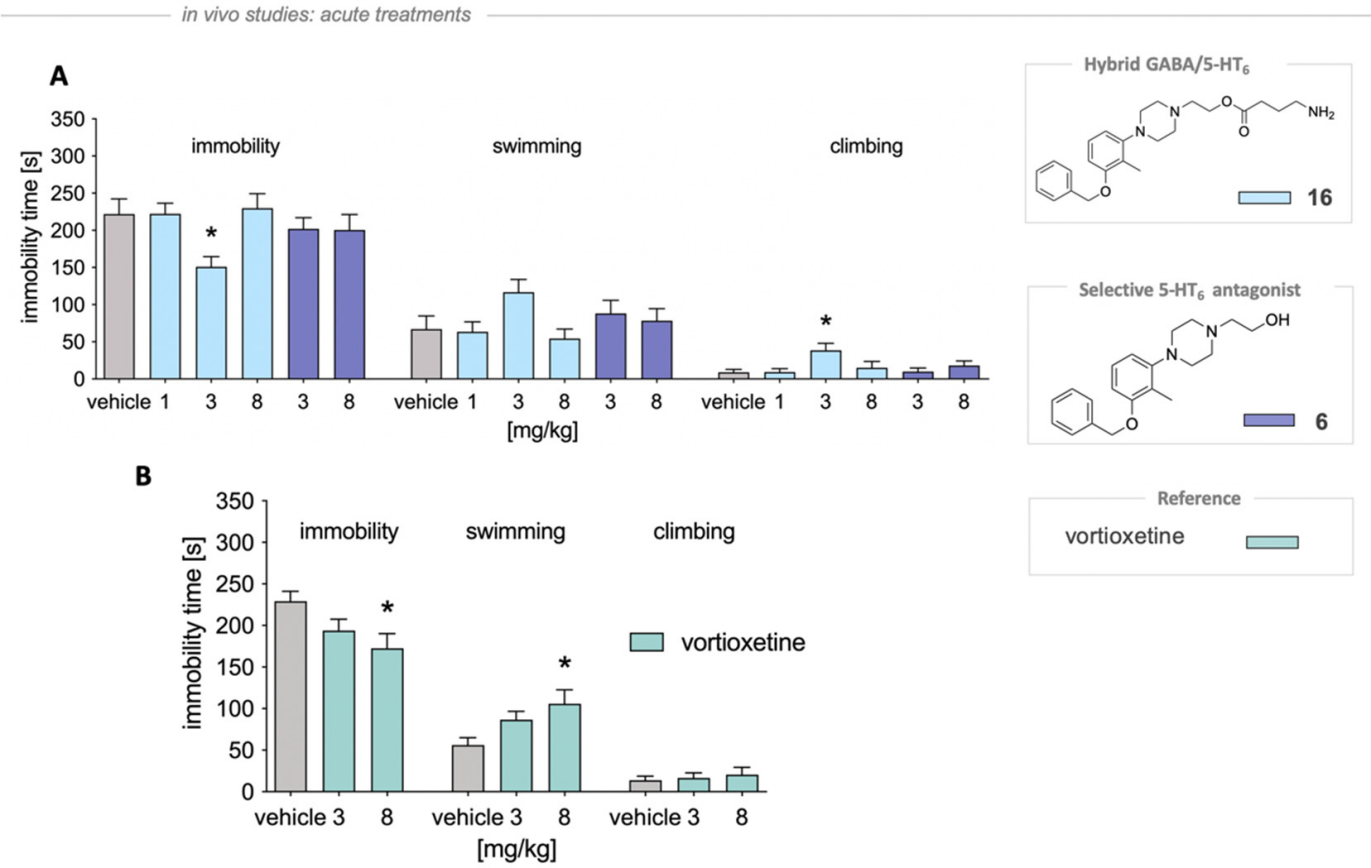
Antidepressant-like properties of **16** in the modified FST in rats. Tested hybrid molecule and vortioxetine were administered ip 30 min prior the test. The 10% cyclodextrin was administered to the vehicle-treated groups. The one-way ANOVA followed by Newman–Keuls post hoc or Student *t* test was used in statistical analysis: *p < 0.05 vs vehicle-treated group, *n* = 7–8 per group.

In the rat FST, selected molecule **16** administrated at 3 mg/kg (but not 1 or 8 mg/kg), in comparison with control group, significantly reduced immobility time by 31.9% (*F*(5,36) = 2.801, *p* < 0.05) and increased climbing behaviors by 336.5% (*F*(5,36) = 3.049, *p* < 0.05), without affecting the duration of swimming (*F*(5,36) = 1.680, *p* = 0.165) (Figure 5A). We observed that the alcohol **6**, carrying solely 5-HT_6_ antagonistic activity, did not influence any of the above behaviors (Figure 5). Alongside, we did not observe any influence of **16** on the animal’s spontaneous locomotor when administered at a dose of 3 mg/kg, indicating that the antidepressant-like activity was specific (Table 3). Vortioxetine, a positive control antidepressant drug, at the dose 8 mg/kg significantly reduced immobility time by 24.7% (*F*(2,20) = 3.538, *p* < 0.05) and prolonged the swimming time by 88.1% (*F*(2,20) = 3.729, *p* < 0.05) but had no impact on the climbing (*F*(2,20) = 0.165, *p* = 0.849) (Figure 5B).

**Figure 6:**
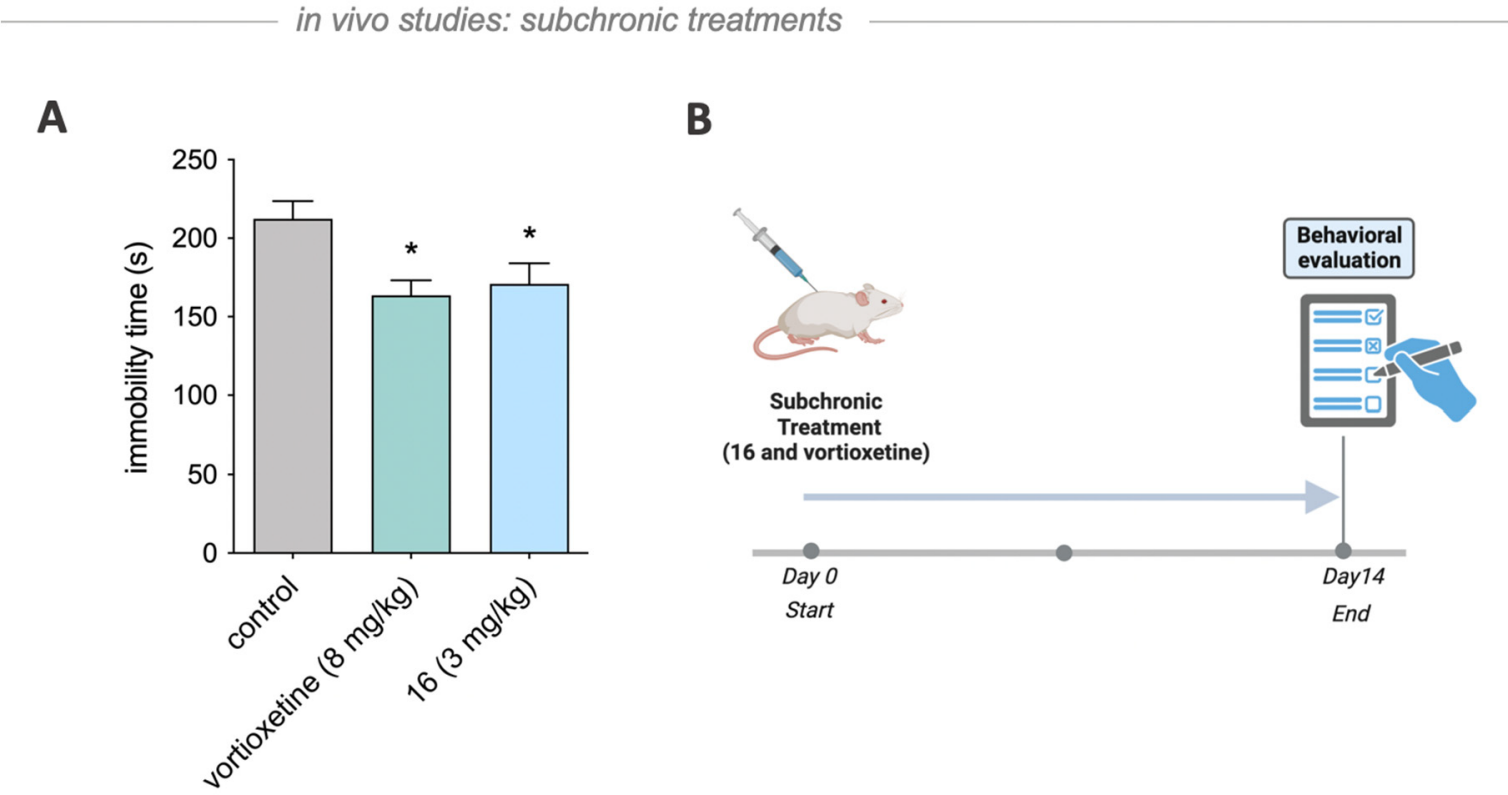
Antidepressant-like effect of **16** after chronic administration in the rat forced swim test. Both vortioxetine and **16** were administered intraperitoneally for 14 consecutive days. Sixty minutes after the last injection the test was performed. Vehicle-treated groups received 10% cyclodextrin for 14 days. Statistical analysis: one-way ANOVA followed by Newman-Keuls post hoc or Student *t* test: *p < 0.05 vs vehicle-treated group; *n* = 10 per group.

The antidepressant-like activity of **16** was also confirmed after subchronic treatment, where **16** was administrated for 14 days at a dose of 3 mg/kg. Subchronic administration of compound **16** at the dose 3 mg/kg and vortioxetine at the dose 8 mg/kg compared with the vehicle-treated group significantly decreased immobility time in rats by 22.9% and 19.5% (*F*(2,27) = 5.330, *p* < 0.05), respectively (Figure 6). Notably, the tested dose of 3 mg/kg of substance **16** did not result in any significant changes in the spontaneous locomotor activity. This indicates that any observed antidepressant-like effect was specific and not due to a general increase in activity levels (Table 4). These findings correspond with our previous reports^22^ on antidepressant-like properties of dually acting GABA-A agonist and 5-HT_6_ antagonist hybrid molecules and, within the present study, have been confirmed for the novel chemotype.

**Table 4:** Open Field Test in Rats Showing the Influence of Acute and Subchronic **16** and Vortioxetine Administration on the Locomotor Activity

treatment	dose	total distance traveled			rearing			*X* ambulation			*Y* ambulation		
Acute Treatment^a^													
vehicle		485.3	±	43.0	29.0	±	2.0	187.5	±	22.0	190.2	±	18.7
**16**	3	506.3	±	36.7	34.5	±	5.4	221.3	±	17.9	213.7	±	18.5
Chronic Treatment^b^													
vehicle		2467	±	142	53.8	±	3.4	246.4	±	14.7	243.1	±	18.8
**16**	3	2575	±	92	52.2	±	2.9	260.4	±	15.0	255.3	±	12.3
vortioxetine	8	2753	±	120	50.6	±	5.4	260.1	±	10.4	265.9	±	10.4

a The compounds were administered ip 30 min prior the experiments. Vehicle-treated groups: 10% cyclodextrin (ip). Statistical analysis: one-way ANOVA followed by Newman–Keuls post hoc or Student *t* test. *n* = 6 per group.

b Both vortioxetine and **16** were administered intraperitoneally for 14 consecutive days. Sixty minutes after the last injection, the test was performed. Vehicle-treated groups received 10% cyclodextrin for 14 days. Statistical analysis: one-way ANOVA followed by Newman–Keuls post hoc or Student *t* test. *n* = 10 per group.

## 3 Conclusions

Neuroinflammation and impaired GABA-ergic signaling often accompany the pathophysiology of depression, leading to poor clinical outcomes.^11,13^ Interest in this critical issue inspired us to design a set of hybrid compounds that include the GABA molecule and a 5-HT_6_ template that blocks the activity of this receptor. In the present study, we extended our previous research on the GABA-A/5-HT_6_ hybrid molecules by employing the novel “5-HT_6_ antagonists” scaffolds. Among novel chemotypes that were investigated in radioligand binding studies, we chose compound **16**, characterized by high affinity for both targets (5-HT_6_*K*_i_ = 16.0 ± 0.4 nM, GABA-A *K*_i_ = 147.0 ± 12.7). In the physicochemical and ADMET assays **16** displayed favorable drug-like properties and promising chemical stability in rat plasma, brain tissue, and PBS buffer. We also investigated the anti-inflammatory properties of **16** in BV-2 microglia cells, which significantly reduced LPS-stimulated NO, ROS, IL-6, and TNF-α levels. In animal studies, we observed antidepressant-like activities of **16** in the forced swim test after acute administration of **16** at a dose of 3 mg/kg. This activity was maintained following subchronic administration of **16** during 14 consecutive days. Collectively, the present study suggests that combining the dual 5-HT_6_ antagonism and GABA-A receptor agonism could be useful in delivering compounds with anti-inflammatory and antidepressant-like activity. These activities were confirmed for another “5-HT_6_ antagonist” binding chemotype, suggesting the general applicability of this dual strategy. The present findings point to compound **16** as a novel lead for broader studies in the area of depression and coexisting neuroinflammation.

## 4 Methods

### 4.1 Molecular Modeling

The *in silico* studies were performed using SwissADME tool and Small-Molecule Drug Discovery Suite (Schrödinger, Inc.). The first one was used to test the structures of final molecules to detect notorious classes of reactive assay interference compounds (PAINS) and potential toxicophores (Brenk alert). The SMILES strings describing molecules **16**–**20** were verified by the server (http://www.swissadme.ch), showing no alerts. The Small-Molecule Drug Discovery Suite (Schrödinger, Inc.) was used for the docking procedures. The assignment of amino acid residues was performed according to their position in the protein sequence of the GABA-A receptor and Ballesteros–Weinstein nomenclature (5-HT_6_ receptor).^36^ Ligand structures were optimized using the LigPrep tool and the OPLS4 force field. The major ionization forms were calculated at pH 7 ± 2. Docking studies for the 5-HT_6_ receptor were carried out utilizing the 5-HT_6_ receptor model, established on the 7XTB experimental structure, preparation and validation of which were conducted previously.^22,37,38^ The Glide SP flexible docking procedure was applied with the centroid of a grid box and H-bond constraints set on Asp3.32. Docking studies for the GABA-A receptor were performed using the previously developed complex GABA-A receptor with dual GABA-A/5-HT_6_ agonist–compound **3B**, which was prepared based on the previously reported cryo-EM structure of α1β3γ2L GABA-A receptor forming a complex GABA and picrotoxin (PDB code 6HUJ).^22^ The molecules were docked using Glide SP docking, in which the grid was set on the ligand and docking was controlled to its core structure (SMARTS: CCCC[N+]) and Glu-155. The docking scores were surveyed according to the presence of proper molecular interactions, considering values of Glide gscore scoring function.

### 4.2 Synthetic Procedures

#### 4.2.1 General Chemistry Information

The chemical reagents were acquired as reagent grade and employed straightforwardly to the synthesis. Purification of the reaction mixtures was performed using automatic CombiFlash RF (Teledyne Isco) and RediSep R_f_ flash columns (silica gel 60, particle size 40–63 μm) or RediSep Gold (silica gel 60, particle size 20–40 μm). The progress of the reaction was monitored by TLC plates acquired from Merck containing an aluminum foil covered with silica gel 60 F_254_ and visualized with UV light (254 nm). The UPLC–MS purity of all molecules investigated in pharmacological studies was measured to be >95.2%. The UPLC–MS analyses were performed on a Waters ACQUITY UPLC (Waters Corporation, Milford, MA, USA) combined with a Waters tandem quadrupole mass spectrometer (TQD) (electrospray ionization (ESI) mode with TQD). The analytical measurements were conducted on C18 column ACQUITY UPLC BEH with the particle size of 2.1 mm × 100 mm and 1.7 μm particle size. The mobile phase conditions and gradient were the following: 10 min, flow rate = 0.3 mL/min, temperature 40 °C, gradient consisting of 95–0% eluent A that is a solution containing 0.1% formic acid in water (v/v) and eluent B that is 0.1% solution of formic acid in acetonitrile (v/v). 10 μL of each sample was injected, and chromatograms were recorded using a Waters eλ photodiode array detector and analyzed in the range of 200–700 nm with a resolution of 1.2 nm and a sampling rate of 20 points per second. The LCMS spectra were analyzed using Waters MassLynx 4.0 software. The ^1^H NMR, ^13^C NMR, and ^19^F NMR spectra were acquired on the Varian Mercury spectrometer (Varian Inc., Palo Alto, CA, USA) and the FT-NMR 500 MHz JEOL spectrometer (JEOL Ltd., Tokyo, Japan) JNM-ECZR500 RS1, version ECZR, operating at 300 or 500 MHz (^1^H NMR), 75 or 126 MHz (^13^C NMR), and 282 or 471 MHz (^19^F NMR), respectively. Chemical shifts δ are reported in ppm and analyzed adjusting the ppm value, first according to the appropriate reference solvents: chloroform-*d*, methanol-*d*_4_, or DMSO-*d*_6_. The signal multiplicities are described using the commonly found abbreviations: s (singlet), d (doublet), t (triplet), q (quartet), m (multiplet), br s (broad singlet), bd (broad doublet), etc. All the acquired spectra were analyzed using the ACD/Spectrus Procesor 2017.

#### 4.2.2 Preparation of Key Building Blocks **3** and **5**

##### *tert*-Butyl 4-(2-Oxo-2,3-dihydrobenzo[*d*]oxazol-7-yl)piperazine-1-carboxylate (**b**)

To a solution of 7-(piperazin-1-yl)benzo[*d*]oxazol-2(3*H*)-one (7.05 mmol, 1.8 g, 1 equiv) in DCM (35 mL), Boc anhydride (7,76 mmol, 11.7 g, 1.1 equiv) and triethylamine (12 mmol, 1.7 mL, 1.7 equiv) were added, and the reaction was stirred at room temperature for 12 h. Next, DCM was evaporated and the crude mixture was purified by column chromatography using Hex:EtOAc 6:4 as eluent. The final molecule was afforded as crystallizing oil, yield 54%. ^1^H NMR (chloroform-*d*, 500 MHz) δ 9.71 (s, 1H), 7.0–7.1 (m, 1H), 6.7–6.7 (m, 1H), 6.6–6.6 (m, 1H), 3.6–3.6 (m, 4H), 3.2–3.3 (m, 4H), 1.4–1.5 (m, 9H). ^13^C NMR (chloroform-*d*, 126 MHz) δ 155.4, 154.8, 135.8, 134.3, 130.4, 124.8, 110.7, 103.1, 80.1, 49.2, 28.4 (carbons overlap). UPLC–MS (ESI) calcd for C_16_H_21_N_3_O_4_ 320.15 (M + H^+^), found 320.20 (M + H^+^).

##### *tert*-Butyl 4-(3-Benzyl-2-oxo-2,3-dihydrobenzo[*d*]oxazol-7-yl)piperazine-1-carboxylate (**c**)

To a solution of **b** (3.85 mmol, 1.23 g, 1 equiv) in dry THF at 0 °C the 1 M solution of *t*-BuOK (4.62 mmol, 4.62 mL, 1.2 equiv) was added, and the resulting slurry was stirred for 40 min at 0 °C. Next, benzyl bromide (4.62 mmol, 0.503 mL, 1.2 equiv) was added and the reaction mixture was stirred at room temperature for 12 h. After that time THF was removed under reduced pressure and the crude mixture was purified using Hex:EtOAc 6:4 as eluent affording the final compound as crystallizaing oil, yield 46%. ^1^H NMR (chloroform-*d*, 300 MHz) δ 7.3–7.4 (m, 5H), 7.00 (t, 1H, *J* = 1.0 Hz), 6.58 (d, 1H, *J* = 9.4 Hz), 6.4–6.5 (m, 1H), 4.98 (d, 2H, *J* = 1.0 Hz), 3.6–3.6 (m, 4H), 3.2–3.3 (m, 4H), 1.5–1.5 (m, 9H). ^13^C NMR (chloroform-*d*, 75 MHz) δ 154.7, 154.4, 135.7, 134.8, 132.9, 131.8, 128.9, 128.2, 127.6, 124.5, 110.8, 102.0 (aromatic carbons overlap), 80.0, 49.2, 46.1, 28.4. UPLC–MS (ESI) calcd for C_23_H_27_N_3_O_4_ 410.15 (M + H^+^), found 410.20 (M + H^+^).

##### 3-Benzyl-7-(piperazin-1-yl)benzo[*d*]oxazol-2(3*H*)-one (**3**)

To a solution of **2** (1.767 mmol, 0.721g, 1 equiv) in EtOH at 0 °C, acetyl chloride (10.21 mmol, 0.8 mL, 5.7 equiv) was added. The resulting reaction mixture was stirred at 55 °C for 2 h. After that time a white solid was precipitated, washed with hexane (15 mL), dried, and used in the next step without further purification (0.280 g, 52%). ^1^H NMR (DMSO-*d*_6_, 300 MHz) δ 9.43 (s, 1H), 7.2–7.4 (m, 5H), 7.0–7.1 (m, 1H), 6.8–6.8 (m, 1H), 6.72 (d, 1H, *J* = 8.8 Hz), 4.99 (s, 2H), 3.4–3.5 (m, 4H), 3.2–3.2 (m, 4H). ^13^C NMR (75 MHz; DMSO-*d*_6_): δ 153.9, 136.0, 134.6, 132.5, 132.1, 129.2, 128.3, 127.9, 125.0, 111.4, 103.3, 46.2, 45.5, 42.9. UPLC–MS (ESI) calcd for C_18_H_19_N_3_O_2_ 310.15 (M + H^+^), found 310.19 (M + H^+^).

##### 4-Benzyl-8-bromo-3,4-dihydro-2*H*-benzo[*b*][1,4]oxazine (**ii**)

A solution of 8-bromo-3,4-dihydro-2*H*-benzo[*b*][1,4]oxazine (1.63 mmol, 0.350g, 1 equiv) in DMF (10 mL) at 0 °C NaH was added portionwise, and the resulting mixture was stirred for 30 min following by addition of benzyl bromide (1.95 mmol, 0.212 g, 1.2 equiv). Resulting orange slurry was stirred at room temperature for 5 h. After that time, the reaction mixture was poured into water at 0 °C (20 mL), EtOAc (20 mL) was added, and the organic layer was separated. Purification over column chromatography using hexane/EtOAc 9:1 afforded **ii** as crystallizing oil (0.395g, 80%). ^1^H NMR (chloroform-*d*, 300 MHz) δ 7.2–7.5 (m, 3H), 6.91 (dd, 1H, *J* = 2.1, 7.3 Hz), 6.6–6.7 (m, 2H), 4.48 (s, 2H), 4.3–4.4 (m, 3H), 3.4–3.5 (m, 3H). ^13^C NMR (chloroform-*d*, 75 MHz) δ 140.6, 137.6, 136.7, 129.1, 128.8, 128.3, 127.4, 127.0, 122.1, 121.3, 111.6, 110.7, 65.1, 55.0, 47.2. UPLC–MS (ESI) calcd for C_15_H_14_BrNO 304.03 (M + H^+^), found 304.19 (M + H^+^).

##### *tert*-Butyl 4-(4-Benzyl-3,4-dihydro-2*H*-benzo[*b*][1,4]oxazin-8-yl)piperazine-1-carboxylate (**iii**)

A mixture of 4-benzyl-8-bromo-3,4-dihydro-2*H*-benzo[*b*][1,4]oxazine (1.72 mmol, 0.520 g, 1 equiv), Boc-piperazine (3.43 mmol, 0.638 g, 2 equiv), rec-BINAP (0.063 mmol, 0.042g, 0.04 equiv), Pd_2_(dba)_3_ (0.034 mmol 0.031g, 0.02 equiv), *t*-BuONa (2.41 mmol, 0.230g, 1.4 equiv) in toluene (10 mL) in sealted tube was heated at 100 °C for 12 h. After that time the reaction mixture was filtered over Celite, toluene was evaporated, and the crude mixture was filtered over silica gel and the compound was used directly in the next step.

##### 4-(4-Benzyl-3,4-dihydro-2*H*-benzo[*b*][1,4]oxazin-8-yl)piperazin-1-ium Chloride (**5**)

A mixture of *tert*-butyl 4-(4-benzyl-3,4-dihydro-2*H*-benzo[*b*][1,4]oxazin-8-yl)piperazine-1-carboxylate (**iii**) (0.370g) and 1 M HCl/EtOAc (35 mL) was stirred for 6 h. The precipitated solid was washed with Et_2_O (15 mL) affording the final compound as a white solid (0.280 g, 75%). ^1^H NMR (DMSO-*d*_6_, 300 MHz) δ 9.6–9.7 (m, 2H), 7.2–7.4 (m, 5H), 6.6–6.7 (m, 1H), 6.5–6.6 (m, 2H), 4.47 (s, 2H), 4.25 (s, 2H), 3.39 (s, 6H), 3.29 (s, 4H). ^13^C NMR (DMSO-*d*_6_, 75 MHz) δ 138.5, 136.5, 136.5, 129.0, 127.5, 127.4, 121.5, 110.1, 108.3 (aromatic carbons overlap), 64.4, 54.7, 48.1, 47.3, 42.6. UPLC–MS (ESI) calcd for C_19_H_23_N_3_O 310.18 (M + H^+^), found 310.21 (M + H^+^).

#### 4.2.3 General Procedure for the Preparation of Alcohols **6**–**10**

A mixture of bromoethanol (1.2 equiv, 0.73 mmol, 0.060 mL), appropriate building block **a**–**e** (1 equiv, 0.61 mmol), and K_2_CO_3_ (1.2 equiv, 0.73 mmol, 0.105 g) in acetonitrile (15 mL) or dioxane (15 mL) was stirred at reflux overnight, then cooled to room temperature, and K_2_CO_3_ was filtered off. The solvent was removed, and the reaction mixture was purified using column chromatography with an eluent: ethyl acetate/dichloromethane/methanol, 2:7:1 (*v/v/v*).

##### 2-(4-(3-(Benzyloxy)-2-methylphenyl)piperazin-1-yl)ethan-1-ol (**6**)

Crystallizing oil, yield 64%. ^1^H NMR (500 MHz, chloroform-*d*) δ 7.47–7.45 (m, 2H), 7.42–7.38 (m, 2H), 7.34 (d, *J* = 7.2 Hz, 1H), 7.13 (t, *J* = 8.2 Hz, 1H), 6.76–6.68 (m, 2H), 5.09 (s, 2H), 3.69–3.67 (m, 2H), 2.97 (br t, *J* = 4.7 Hz, 4H), 2.79–2.68 (m, 4H), 2.67–2.63 (m, 3H), 2.25 (s, 3H). ^13^C NMR (126 MHz, chloroform-*d*) δ 157.68, 152.50, 137.57, 128.47, 127.68, 127.08, 126.30, 121.18, 111.77, 107.01 (aromatic carbons overlap), 70.05, 59.28, 57.66, 53.32, 51.93, 11.03. UPLC–MS (ESI) calcd for C_20_H_26_N_2_O_2_ 327.24 (M + H^+^), found 327.32 (M + H^+^).

##### 1-Benzyl-4-(4-(2-hydroxyethyl)piperazin-1-yl)-1,3-dihydro-2*H*-benzo[*d*]imidazol-2-one (**7**)

Crystallizing oil, yield 90%. ^1^H NMR (500 MHz, methanol-*d*_4_) δ 7.33–7.20 (m, 5H), 6.96–6.90 (m, 1H), 6.76–6.71 (m, 1H), 6.70–6.65 (m, 1H), 5.05–5.01 (m, 2H), 3.76–3.69 (m, 2H), 3.09–3.00 (m, 4H), 2.82–2.73 (m, 4H), 2.68–2.60 (m, 2H) (NH and OH protons not detected). ^13^C NMR (126 MHz, methanol-*d*_4_) δ 155.53, 136.72, 136.63, 130.81, 128.44, 128.41, 127.32, 127.03, 126.96, 121.78, 120.95, 111.03, 103.80 (aromatic carbons overlap), 59.97, 58.41, 53.30, 50.19, 43.82. UPLC–MS (ESI) calcd for C_20_H_24_N_4_O_2_ 353.19 (M + H^+^), found 353.24 (M + H^+^).

##### 3-Benzyl-7-(4-(2-hydroxyethyl)piperazin-1-yl)benzo[*d*]oxazol-2(3*H*)-one (**8**)

Crystallizing oil, yield 64%. ^1^H NMR (chloroform-*d*, 500 MHz) δ 7.3–7.3 (m, 5H), 6.9–7.0 (m, 1H), 6.6–6.6 (m, 1H), 6.4–6.5 (m, 1H), 4.96 (s, 2H), 3.6–3.7 (m, 2H), 3.3–3.3 (m, 5H), 2.7–2.7 (m, 4H), 2.6–2.6 (m, 2H). ^13^C NMR (75 MHz, chloroform-*d*) δ 155.36, 154.31, 135.63, 134.70, 132.55, 131.57, 128.76, 128.04, 127.40, 124.41, 110.49, 101.56 (aromatic carbons overlap), 64.48, 62.54, 59.34, 57.69, 52.71, 49.00, 45.92. UPLC–MS (ESI) calcd for C_20_H_23_N_3_O_3_ 354.17 (M + H^+^), found 354.19 (M + H^+^).

##### 4-Benzyl-8-(4-(2-hydroxyethyl)piperazin-1-yl)-2*H*-benzo[*b*][1,4]oxazin-3(4*H*)-one (**9**)

Crystallizing oil, yield 62%. ^1^H NMR (300 MHz, DMSO-*d*_6_) δ 7.35–7.18 (m, 5H), 6.87–6.79 (m, 1H), 6.66–6.60 (m, 2H), 5.14–5.09 (m, 2H), 4.76–4.71 (m, 2H), 3.52–3.47 (m, 4H), 3.39 (s, 1H), 3.02–2.92 (m, 4H), 2.56–2.53 (m, 2H), 2.46–2.39 (m, 2H). ^13^C NMR (75 MHz, DMSO-*d*_6_) δ 165.09, 141.97, 137.97, 136.96, 129.82, 128.97, 127.45, 126.86, 122.82, 113.63, 109.95 (aromatic carbons overlap), 67.31, 63.05, 60.59, 58.76, 53.67, 50.49, 48.9, 44.22. UPLC–MS (ESI) calcd for C_21_H_25_N_3_O_3_ 368.19 (M + H^+^), found 368.23 (M + H^+^).

##### 2-(4-(4-Benzyl-3,4-dihydro-2*H*-benzo[*b*][1,4]oxazin-8-yl)piperazin-1-yl)ethan-1-ol (**10**)

Crystallizing oil, yield 29%. ^1^H NMR (chloroform-*d*, 300 MHz) δ 7.2–7.4 (m, 5H), 6.6–6.8 (m, 1H), 6.3–6.5 (m, 2H), 4.44 (s, 2H), 4.3–4.4 (m, 2H), 3.6–3.7 (m, 2H), 3.3–3.4 (m, 2H), 3.09 (m, 4H), 2.72 (br t, 4H, *J* = 4.4 Hz), 2.62 (t, 2H, *J* = 5.3 Hz), 2.35 (br s, 1H). ^13^C NMR (chloroform-*d*, 75 MHz) δ 154.8, 140.9, 138.2, 136.5, 136.2, 128.6, 127.1, 127.0, 121.0, 108.2, 107.9, 79.6, 64.4, 55.5, 50.9, 47.1 (aliphatic carbons overlap), 28.5. UPLC–MS (ESI) calcd for C_21_H_27_N_3_O_2_ 354.21 (M + H^+^), found 354.27 (M + H^+^).

#### 4.2.4 General Procedure for the Preparation of Boc-Intermediates **11**–**15** and Final Compounds **16**–**20**

To a solution of appropriate alcohol **6**–**10** (1 equiv, 0.6 mmol) in dry DCM (10 mL), Boc-GABA (1.1 equiv, 0.72 mmol, 0.147 g), DCC (1 equiv, 0.72 mmol, 0.148 g), DMAP (0.1 equiv, 0.06 mmol, 0.007 g) were added, and the resulting reaction mixture was stirred for 12 h. Next, the solid was separated from the reaction mixture and the filtrate was evaporated. Boc intermediates **11**–**15** were used straightforwardly in the subsequent step without purification. Thus, a mixture of Boc intermediate (0.5 mmol) and 1 M solution of HCl in EtOAc (30 mL) was stirred for 12 h at room temperature. The resulting solid was filtered off, washed with EtOAc (20 mL), and dried.

##### 1-(2-((4-Ammoniobutanoyl)oxy)ethyl)-4-(3-(benzyloxy)-2-methylphenyl)piperazin-1-ium Dichloride (**16**)

White solid, yield 52%. ^1^H NMR (300 MHz, DMSO-*d*_6_) δ 11.60–11.43 (br s, 1H), 8.10–7.97 (br s, 3H), 7.54–7.26 (m, 5H), 7.22–7.10 (m, 1H), 6.91–6.78 (m, 1H), 6.73–6.62 (m, 1H), 5.16–5.03 (m, 2H), 4.59–4.39 (m, 2H), 3.63–3.38 (m, 4H), 3.18–3.12 (m, 2H), 2.81–2.70 (m, 2H), 2.59–2.52 (m, 2H), 2.41 (t, *J* = 7.2 Hz, 2H), 2.15 (s, 3H), 1.94–1.62 (m, 4H). ^13^C NMR (75 MHz, DMSO-*d*_6_) δ 172.34, 171.52, 157.55, 151.29, 137.88, 128.88, 128.13, 127.74, 127.06, 120.53, 112.00, 108.35, 69.82, 52.31, 48.72, 38.41, 37.02, 35.28, 30.73, 29.79, 23.02, 22.58, 11.24. UPLC–MS (ESI) calcd for C_24_H_33_N_3_O_3_ 412.25 (M + H^+^), found 412.35 (M + H^+^).

##### 1-(2-((4-Ammoniobutanoyl)oxy)ethyl)-4-(1-benzyl-2-oxo-2,3-dihydro-1*H*-benzo[*d*]imidazol-4-yl)piperazin-1-ium Dichloride (**17**)

White solid, yield 26%. ^1^H NMR (300 MHz, DMSO-*d*_6_) δ 10.92 (br s, 3H), 7.35–7.21 (m, 4H), 6.92–6.76 (m, 3H), 6.69 (d, *J* = 7.9 Hz, 1H), 6.60 (d, *J* = 8.2 Hz, 1H), 4.97 (s, 2H), 4.15 (br t, *J* = 5.8 Hz, 2H), 3.02–2.90 (m, 8H), 2.69–2.57 (m, 4H), 2.27 (td, *J* = 7.4, 12.3 Hz, 2H), 1.70–1.51 (m, 2H). ^13^C NMR (75 MHz, DMSO-*d*_6_) δ 173.05, 156.03, 154.84, 137.77, 136.62, 130.98, 128.99, 127.75, 127.69, 121.54, 120.74, 110.59, 103.33 (aromatic carbons overlaps), 61.67, 56.58, 53.34, 50.51, 43.69, 35.22, 31.39, 25.37. UPLC–MS (ESI) calcd for C_24_H_31_N_5_O_3_ 437.24 (M + H^+^), found 437.31 (M + H^+^).

##### 1-(2-((4-Ammoniobutanoyl)oxy)ethyl)-4-(3-benzyl-2-oxo-2,3-dihydrobenzo[*d*]oxazol-7-yl)piperazin-1-ium Dichloride (**18**)

White solid, yield 43%. ^1^H NMR (500 MHz, methanol-*d*_4_) δ 7.27–7.18 (m, 5H), 7.05–6.96 (m, 1H), 6.73–6.60 (m, 2H), 4.92 (s, 2H), 4.52–4.38 (m, 2H), 3.89–3.76 (m, 2H), 3.73–3.60 (m, 2H), 3.55–3.44 (m, 2H), 3.38–3.28 (m, 4H), 3.00–2.86 (m, 2H), 2.63–2.50 (m, 2H), 1.98–1.78 (m, 2H). ^13^C NMR (126 MHz, methanol-*d*_4_) δ 172.23, 154.49, 135.33, 133.77, 133.06, 131.94, 128.66, 127.93, 127.46, 124.81, 111.56, 103.49 (aromatic carbons overlap), 58.51, 55.98, 52.83, 46.62, 45.64, 39.34, 30.81, 22.43. UPLC–MS (ESI) calcd for C_24_H_30_N_4_O_4_ 439.23 (M + H^+^), found 439.42 (M + H^+^).

##### 1-(2-((4-Ammoniobutanoyl)oxy)ethyl)-4-(4-benzyl-3-oxo-3,4-dihydro-2*H*-benzo[*b*][1,4]oxazin-8-yl)piperazin-1-ium Dichloride (19)

White solid, yield 68%. ^1^H NMR (500 MHz, methanol-*d*_4_) δ 7.34–7.09 (m, 5H), 6.91–6.63 (m, 3H), 5.14 (s, 2H), 4.72 (s, 2H), 3.98–3.84 (m, 2H), 3.79–3.45 (m, 4), 3.06–2.90 (6H), 2.41–2.33 (m, 2H), 2.00–1.81 (m, 4H). ^13^C NMR (126 MHz, methanol-*d*_4_) δ 173.15, 164.36, 139.31, 138.24, 136.18, 128.46, 127.07, 126.37, 122.65, 114.83, 114.10, 111.45 (aromatic carbons overlap), 67.42, 58.80, 58.63, 55.27, 52.41, 44.45, 39.30, 36.62, 34.63, 30.01, 22.52. UPLC–MS (ESI) calcd for C_25_H_32_N_4_O_4_ 453.24 (M + H^+^), found 453.31 (M + H^+^).

##### 1-(2-((4-Ammoniobutanoyl)oxy)ethyl)-4-(4-benzyl-3,4-dihydro-2*H*-benzo[*b*][1,4]oxazin-8-yl)piperazin-1-ium Dichloride (20)

White solid, yield 41%. ^1^H NMR (300 MHz, DMSO-*d*_6_) δ 11.41 (br s, 1H), 8.36–8.28 (s, 1H), 8.15–8.03 (br s, 2H), 7.38–7.17 (m, 5H), 6.69–6.57 (m, 1H), 6.46–6.36 (m, 1H), 6.31–6.17 (m, 1H), 4.60–4.37 (m, 4H), 4.31–4.13 (m, 2H), 3.32–2.90 (m, 10H), 2.91–2.70 (m, 4H), 2.59–2.51 (m, 2H), 1.93–1.76 (m, 2H). ^13^C NMR (75 MHz, DMSO-*d*_6_) δ 172.38, 138.95, 138.95, 136.34, 136.23, 136.23, 128.92, 127.49, 127.29, 121.17, 107.46 (aromatic carbons overlap), 79.70, 64.30, 54.73, 52.39, 47.50, 47.41, 38.42, 33.78, 30.76, 22.62. UPLC–MS (ESI) calcd for C_25_H_34_N_4_O_3_ 439.26 (M + H^+^), found 439.45 (M + H^+^).

### 4.3 Radioligand Binding Studies and Functional Assays

#### 4.3.1 Determination of Affinity for GABA-A Receptor

The study was acquired according to the earlier protocols.^39^ The rats’ brains were homogenized and prepared accurately according to the previous protocol.^39^ On the day of the study, brain homogenates were thawed at room temperature and “diluted” in 20 volumes of ice-cold 50 mM Tris-HCl buffer (pH 7.4) and centrifuged (20.000*g*, 30 min, 0–4 °C). The assay was performed directly on 96-well microplates, which contained 50 mM Tris-HCl buffer (pH 7.4) in a total volume of 300 μL. Reaction mix included 240 μL of the brain tissue suspension, 30 μL of [^3^H]-muscimol, and 30 μL solution of tested compounds (administrated at various concentrations: 10^–10^–10^–5^ M). In order to determine potential nonspecific binding, 100 μM GABA was added. The 96-well microplates containing the reaction mix were incubated for 10 min at 0 °C, followed by rapid filtration over glass fiber filters FilterMate B (PerkinElmer, USA) using the Harvester-96 MACH III FM (Tomtec, USA). The filter mats were dried using a microwave and then placed inside a plastic bag (PerkinElmer, USA). After that, they were soaked in 10 mL of Ultima Gold MV liquid scintillation cocktail (PerkinElmer, USA). Subsequently, the radioactivity present on the filter was quantified using a MicroBeta TriLux 1450 scintillation counter (PerkinElmer, USA). *K*_i_ values were estimated according to the Cheng and Prusoff equation. The study was performed in duplicates. The statistical analysis was performed using (GraphPad Prism, version 4.0, San Diego, CA, USA).

#### 4.3.2 Radioligand Binding Assays for 5-HT_6_ Receptors

To determine the affinity for 5-HT_6_, the cryopreserved membranes from HEK-293 cells stably transfected with the human recombinant 5-HT_6_ receptor were used.^40^ Preparation of the tested compounds included preparation of stock solutions in DMSO (1 mM) and subsequent serial dilutions (10^–10^–10^–5^ M) of tested compounds in the appropriate buffer (50 mM Tris buffer, pH 7.4, 10 mM MgCl_2_, 0.5 mM ethylenediaminetetraacetic acid). The serial dilutions were acquired directly on a 96-well microplate. The reaction mix included 50 μL of appropriate concentration of tested molecule, 50 μL of [^3^H]LSD 2.5/2.0 nM, and 150 μL of diluted membranes. The microplate containing the reaction mix was protected with a sealing tape and incubated for 60 min at 37 °C. Quenching of the reaction mixtire included: rapid filtration through UniFilter 96 GF/B filter microplate and rapid washes using 200 μL 50 mM assay buffer and vacuum manifold and 96-well pipettor. The collected microplates were dried overnight at 37 °C. The UniFilter bottoms were sealed, and then 30 μL of Betaplate Scint liquid scintillator (PerkinElmer) was added to each well. The radioactivity was quantified using a MicroBeta TriLux 1450 scintillation counter (PerkinElmer) with an estimated efficiency of 30%. The obtained data were fit to a one-site curve-fitting equation using Prism 5 software (GraphPad Software), and the *K*_i_ values were determined using the Cheng–Prusoff equation. Radioligand binding was performed in duplicates.

#### 4.3.3 Functional Assays for 5-HT_6_ Receptor

The studies were acquired according to previous protocols.^41^ All the examined compounds (novel and the reference) were dissolved in DMSO (1 mM) and diluted accordingly using the assay buffer, directly in 96-well microplate. The recombinant CHO-K1 cells expressing human GPCR, mitochondrially targeted aequorin, and the promiscuous G protein α16 specific for 5-HT_6_ were used for the functional assay. The cells were thawed and suspended in the appropriate buffer containing DMEM/HAM’s F12 with 0.1% protease free BSA and were then centrifuged. After adding coelenterazine h to a final concentration of 5 μM, the cell pellet was resuspended in the buffer. The cell suspension was incubated at 16 °C for 16 h while being protected from light, gently shaken, and diluted with assay buffer to a concentration of 5000 cells/mL. Following that, 50 μL of the cell suspension was introduced to preloaded white opaque 96-well microplates containing the tested molecules. This was done using the automatic injectors integrated into the radiometric and luminescence plate counter MicroBeta2 LumiJET (PerkinElmer, USA). The light emission resulting from calcium mobilization was monitored for a duration of 60 s. For examination of the antagonistic properties of tested molecule, following 30 min of incubation, the reference agonist was administrated and the light emission was detected.

#### 4.3.4 Electrophysiological Studies

Electrophysiological studies were acquired using the QPatch16X automatic patch clamp platform (Sophion Biosciences) as described before.^42,43^ The HEK293 cells stably expressing the human α1β3γ2 GABA-A receptor were used. Before the assay, cells were detached from the culture flask by treating them with TrypLE Express solution (Life Technologies) and subsequently suspended in serum-free media. The cells were loaded into a 1.5 mL microtube on the automated electrophysiology instrument, which subsequently performed a spin-down using its built-in centrifuge. Following this, the cells were washed with Ringer’s extracellular solution. The cells were moved to the pipetting wells of a 16-channel planar patch chip plate (QPlate 16X) designed for single-use. A combined suction/voltage protocol was then applied to establish gigaseals. Subsequent suction resulted in the establishment of whole-cell configuration. The chloride currents flowing through the GABA-A receptor were quantified for a duration of 7 s following the administration of the test molecule. During the entire whole-cell recording, the holding potential was maintained at −90 mV. The extracellular solution consisted of 2 mM 4KCl, 145 mM NaCl, 10 mM HEPES, CaCl_2_, 1 mM MgCl_2_, 10 mM glucose (pH 7.4, 300 mOsm). The intracellular solution contained 140 mM CsF, 1 mM EGTA, 5 mM CsOH, 10 mM HEPES, 20 mMNaCl (pH 7.2, 320 mOsm). The assay was configured within the instrument software in a sequence that involved the following steps: administration of 10 μM GABA (reference agonist AG1); administration of 1 μM test molecule (T1); coadministration of 1 μM test molecule and 10 μM GABA (T2); a second application of 10 μM GABA (AG2); and introduction of 10 μM bicuculline (reference antagonist) together with 10 μM GABA (ATG). The acquired data were examined with QPatch assay software (v5.0, Sophion Biosciences)^44^ and are represented as the mean of three separated measurements performed on distinct cells. The efficacy of the tested molecules was determined by calculating the baseline-corrected ratio of the maximum current amplitudes elicited by the tested compounds and the reference agonist (T1-ATG/AG1-ATG or T2-ATG/AG1-ATG). The raw current recordings were standardized and presented as a percentage of the current amplitude evoked by the reference agonist, using the QPatch assay software (v5.0, Sophion Biosciences).

### 4.4 Physicochemical and ADMET Assays

#### 4.4.1 Thermodynamic Solubility in PBS and Stability Assays

The quantitative HPLC analyses were acquired using Waters Alliance e2695 separations module (Waters, Milford, CT, USA) containing 2998 photodiode array (PDA), a detector (Waters, Milford, CT, USA), and the SpeedROD RP-18e 50–4.6 mm column (Merck, KGaA, Darmstadt, Germany). The temperature of the column was preserved at 30 °C. The experiment was conducted under the following conditions: a flow rate of 5 mL/min, eluent A (water/0.1% HCOOH), eluent B (MeCN/0.1% HCOOH), a gradient of starting from 0% of B to 100% of B over a duration of 3 min. Each sample was injected at a volume of 10 μL in triplicate. *Thermodynamic solubility in PBS measurement:* The chromatograms were examined at 255 nm (perphenazine) and 212 nm (**16**). Stock solutions of analyzed compounds (**16** and reference) were dissolved in methanol to achieve the concentration of 1 mg/mL. The stock solutions were mixed with methanol and diluted, resulting in various solutions with concentrations ranging from 1.0 to 0.125 μg/mL. These solutions were then utilized to generate calibration curves by plotting AUC versus concentration in μg/mL. The examined molecules (2 mg) were dissolved in 1 mL of Dulbecco’s phosphate buffered saline (DPBS). The mixture was then continuously agitated at 22 °C for 24 h using a thermoshaker. Following this period, the mixtures were filtered through a cellulose acetate syringe filter (with a pore size of 0.22 μm), moved to a chromatographic vial, and analyzed. To quantify the investigated compounds, the areas beneath their respective peaks on DAD chromatograms were employed. Solubility was calculated using the calibration curves. *Chemical stability assay:* Stock solution of **16** in DMSO (10 mg/mL) was used. Next, 25 μL of stock solution was mixed with 975 μL of PBS (Dulbecco’s phosphate-buffered saline, Sigma, Poland). The mix was gently stirred at 22 °C. HPLC analyses were used to quantify the percentage of molecules that remained at each time point relative to the 0 min time point. *Brain tissue stability assay:* Brain homogenate was prepared according to a previous protocol.^22^ Compound **16** was dissolved in DMSO to achieve the concentration of 5 mg/mL. Then, 50 μL of the stock solution was combined with 50 μL of ice-cold 50 mM Tris-HCl buffer (pH 7.4) and 150 μL of brain homogenate (suspended in 20 volumes of 50 mM Tris-HCl buffer, ice cold, pH 7.4). The mix was then incubated at 37 °C and quenched at several time points. To terminate the reaction mixture, 250 μL of the appropriate reaction mixture was mixed with 1000 μL of MeCM in the Eppendorf Tube. Following 10 min of shaking (1500 rpm), samples were centrifuged (10 000 rpm, 10 min, 4 °C) and the acquired supernatant was collected. Before conducting HPLC analysis, the supernatant was filtered via a cellulose acetate syringe filter with a pore size of 0.45 μm and then transferred to a chromatographic vial. HPLC analysis was used to determine the percentage of the tested molecule remaining at each time point, relative to the 0 min time point. *Plasma stability assay* was performed as described previously.^45^ Frozen rat plasma (Wistar) was thawed. Next, 4 μL of stock solution of tested compound **16** in DMSO (20 mg/mL) was mixed with 396 μL of rat plasma and incubated at 37 °C. The samples were collected at relative time points. The reaction was terminated by adding 1200 of μL acetonitrile/methanol mixture (50:50, v/v). The samples were centrifuge at 25 000 rpm for 10 min. HPLC analysis was used to determine the percentage of the tested molecule remaining at each time point relative to the 0 min time point. All the experiments were performed in duplicate.

#### 4.4.2 Metabolic Stability

The assay was performed by Eurofins Discovery, a contract research organization, according to standard methodology, described previously.^46^

#### 4.4.3 Permeability

To estimate the passive transport through cell membranes, we used specialized PAMPA Plate System Gentest acquired from Corning (Tewksbury, MA, USA). The assay was performed as described in the manufacturer’s protocol. The quantitative measurements of tested molecules in apical and basolateral wells were conducted using LC/MS (Waters ACQUITY TQD system with the TQ detector, Milford, USA) and a specific internal standard. The *P*_e_ permeability coefficient was calculated rendering the formulas described previously^47,47^ and compared to the reference compound sulpiride.

#### 4.4.4 Hepatotoxicity Assay

Hepatotoxiticy assay was conducted according to our previously described procedures.^42^*Cell culture and treatment*: The human hepatocellular carcinoma (HepG2) cell line was acquired from ATCC, Manassas, USA (HB-8065), and maintained according to the manufacturer’s ATCC protocol. Cell culture conditions included a medium consisting of Dulbecco’s modified Eagle’s medium (DMEM, Merck), 10% fetal bovine serum, 100 mg/mL streptomycin, 100 IU/mL penicillin (acquired from ThermoFisher), humidified atmosphere containing 5% of carbon dioxide, temperature of 37 °C. The stock solution of the tested compound was prepared in DMSO (10 mM) and next diluted with buffered phosphate saline (PBS, Merck). Cell membrane damage was examined with the use of a ToxiLight bioassay (Lonza), rendering the manufacturer’s protocol described previously.^42^ A culture medium containing 10% Triton X-100 (Merck) was used as the positive control of cell damage, while the negative control contained solely vehicle. After incubating for 5 min, the luminescence was examined with the plate reader POLARstar Omega (BMG Labtech). The results were expressed as a percentage of the positive control, which was defined as the percentage of dead cells relative to the control sample. *Cell viability assay:* The viability of cells was examined with the use of the PrestoBlue reagent (ThermoFisher) and protocol provided by the manufacturer. After incubating for 24 h with the examined compounds, one-tenth of the remaining medium volume was mixed with the reagent (PrestoBlue) in a microplate well. Following incubation at 37 °C during 15 min, the fluorescence (EX 530; EM 580 nm) was measured using the plate reader POLARstar Omega, (BMG Labtech). The viability values obtained are presented as a percentage of live cells relative to the DMSO (control sample). The GraphPad program was used to perform the statistical analysis. The mean values with their corresponding coefficient of variation (CV) were reported for all data. The differences between the groups were analyzed using one-way analysis of variance (ANOVA) followed by Dunnett’s multiple comparison tests as post hoc analysis. The statistical significance: *p* < 0.05.

### 4.5 *In Vitro* Anti-Inflammatory Assessment

#### 4.5.1 Preparation of Cells

Mouse microglial (BV-2) cell line was a generous gift from Prof. Bożena Kamińska-Kaczmarek of the Nencki Institute of Experimental Biology, Polish Academy of Sciences, Warsaw, Poland. The cell culture medium: Dulbecco’s modified with high glucose (DMEM, Glutamx ThermoFisher) and supplemented with 10% inactivated fetal bovine serum heat (ThermoFisher), 100 IU/mL penicillin (Merck) and 100 μg/mL streptomycin (Merck). Cell culture conditions: culture flasks (area 175 cm^2^, Nunc), temperature of 37 °C, 5% CO_2_. For the assessment of the effect of tested compounds on the quantities of reactive oxygen species, nitric oxide, and inflammatory markers, the BV-2 cells were positioned in a 96-well culture plate (5 × 10^4^ cells per well, Falcon). In the case of cell membrane damage assay, a 96-well culture plate (2 × 10^4^ cells per well, Falcon) was used. The cells were cultured in the incubator (37 °C, 5% CO_2_) for 24 h prior to the assay. The tested compounds were dissolved in DMSO to achieve the concentration of 10^–2^ M. Serial dilutions of the compounds were prepared in PBS and added directly to the medium containing adherent cells. The resulting mixtures were examined for any evidence of precipitation or opalescence before the assay. BV-2 cells were incubated with selected molecules (**6**, **16** at a concentration of 10 μM) during 1 h. Subsequently, lipopolysaccharide (100 ng/mL) was added and the resulting mixture was incubated for 18 h. Next, the culture supernatant was collected to measure the levels of nitric oxide (NO), reactive oxygen species (ROS), IL-6, and TNF-α according to the procedures described in detail below. All experiments were performed in duplicates, in three independent experiments.

#### 4.5.2 NO Measurement

The quantities of NO produced by the cells were determined using DAN reagent (2,3-diaminonaphthalene), rendering the method protocol described by Nussler et al.^48^ After incubating at room temperature for 15 min, the fluorescence intensity (EX 360; EM 440 nm) was measured using a microplate reader POLARstar Omega from BMG Labtech. The amounts of NO were then calculated as a percentage of the control (which represents the maximal response of LPS). *ROS measurement.* The fluorescent dye 2,7-dichlorodihydrofluorescein diacetate (DCFH-DA)^49^ was utilized to determine the quantity of ROS released by BV-2 cells. After a 30 min incubation period at 37 °C with 10 μM DCFH-DA, the fluorescence intensity (EX 490; EM 520 nm) was examined using a microplate reader POLARstar Omega from BMG Labtech. The amounts of ROS were then expressed as a percentage of the control (which represents the maximal response of LPS). *The IL-6 and TNF-α levels* detected in the culture supernatants were quantified using LANCE Ultra TR-FRET detection kit (PerkinElmer), rendering the manufacturer’s procedure. The detection of IL-6 and TNF-α was performed independently in a 384-well plate according to the instructions provided. To each well of the plate, 15 μL of sample was added followed by the addition of 5 μL of premixed antibody solution. After incubating IL-6 for 1 h and TNF-α for 3 h in the absence of light, at 22 °C, the plates were transferred to an EnVision plate reader (PerkinElmer). Measurements were conducted at 320 nm wavelength for excitation, at 615 nm for donor emission, and at 660 nm for acceptor emission. The final data were determined as the ratio of the 660 nm signal to the 615 nm signal. The results represent the levels of each cytokine determined as a percentage of control (considered as maximal cell response to LPS). The GraphPad program was used to conduct statistical analysis, with all outcomes presented as mean values with standard deviation (SD). Differences between groups were assessed using one-way ANOVA, followed by post hoc analysis (Dunnett’s multiple comparison tests). Results were considered statistically significant if the *p*-value was less than 0.05. *Evaluation of microglia morphology:* BV-2 cell line was pretreated with the tested compounds (10 μM) and incubated for 1 h, followed by addition of LPS (100 ng/mL). The mixture was incubated during 18 h period of time. Next, the following fluorescent dyes were used: Calcein AM (ThermoFisher), Hoechst 33342 (ThermoFisher), and MitoTracker (ThermoFisher) as indicated previously.^22^ The images were taken with Leica DMI8 microscopy.

### 4.6 Behavioral Evaluation

#### 4.6.1 Animals

Naïve female Wistar rats (weighing 180–200 g) were used in the study. The animals were housed in groups of three to four in standard Makrolon cages (37 cm × 21 cm × 15 cm) under strictly controlled laboratory conditions (ambient temperature 21–24 °C, relative humidity 45–65%) with a 12/12 h light–dark regime (light on at 6:00 a.m. and off at 6:00 p.m.). Animals had unrestricted access to tap water and food (typical pellets). Prior the experiment the animals were randomly appointed to the group. The behavioral studies were accomplished between 8:00 a.m. and 5:00 p.m. with the researcher blind to the study. The experiments were performed according to protocols approved by the Local Ethical Committee in Krakow (Approval Number 147/2018), Poland. Housing and experimental procedures were conducted under the European Union Directive of September 22, 2010 (2010/63/EU) and Polish legislation regarding animal studies. All efforts were made to minimize animal suffering and the number of animals used in the study.

#### 4.6.2 Drugs

Vortioxetine (Sigma, Germany) and the tested compounds (**16** and **6**) were dissolved in 10% cyclodextrin and administered ip 30 min before the test or during 14 consecutive days, with the last injection 60 min before the test. 10% cyclodextrin was administrated to control group.

#### 4.6.3 Forced Swim Test in Rats

A modified forced swim test was carried out according to Detke et al.^50,51^ First, the animals were individually placed inside Plexiglas cylinders, which were 40 cm high and 18 cm in diameter, filled with water at a temperature of 23–25 °C for 15 min. Afterward, the rats were transferred to a Plexiglas box and kept under a 60 W bulb for 30 min to dry. The day after, exactly 24 h later, the rats were once again placed in the cylinder, and their duration of immobility, swimming, and climbing was observed and recorded during a 5 min. The swimming behavior involved energetic movements such as horizontal swimming around the cylinder, while climbing activity referred to upward movements of the forepaws along the swim chamber’s side. Immobility was recorded when the rat only made necessary movements to keep its head above the water. For each test, a fresh water was provided. *Open field test in rats:* The test was conducted as previously reported by our research group.^52^ The test was performed in a darkened room employing the Motor Monitor System (Campden Instruments, Ltd., U.K.). The system was equipped with two SmartFrame Open Field stations, each measuring 40 cm × 40 cm × 38 cm and consisting of 16 × 16 beams. These stations were placed inside sound-attenuating chambers and connected to a PC software using Motor Monitor System (Campden Instruments, Ltd., U.K.), equipped with two SmartFrame Open Field stations (40 cm × 40 cm × 38 cm) with 16 × 16 beams, placed in sound-attenuating chambers, which was linked to a PC software. The animals, belonging to either the vehicle-injected or drug-injected group, were individually positioned at the center of the station. The Motor Monitor System, which was automated, recorded the animal’s ambulation in both the *X* and *Y* axes, as well as the number of rearing and peeping episodes. Additionally, the total distance traveled by each animal during the 5 min test period was also recorded. *Statistical analysis:* Results are presented as the mean ± SEM values. They were estimated using one-way analysis of variance (ANOVA), followed by Newman–Keuls post hoc or unpaired two-tailed Student *t* test. Statistical significance was considered when *p* < 0.05 for differences between groups.

## Supporting Information Available

^1^H NMR, ^13^C NMR, COSY, and LCMS/MS spectra of selected final compounds, predicted binding modes of selected molecules, and hepatotoxicity assay (PDF)
