# Supplementary material for: Dual Molecules Targeting 5-HT6 and GABA-A Receptors as a New Approach to Combat Depression Associated with Neuroinflammation
Source: ACS Chem Neurosci. 2023 Apr 4;14(8):1474–89. doi: 10.1021/acschemneuro.3c00033 (PMC10119930; doi:10.1021/acschemneuro.3c00033)
Supplement: Supplementary file 1 — cn3c00033_si_001.pdf [file cn3c00033_si_001.pdf]

Supporting Information

Dual molecules targeting 5-HT_6 and GABA-A receptors as a new approach to combat depression associated with neuroinflammation

Monika Marcinkowska,*[a] Barbara Mordyl,[a] Agata Siwek,[a] Monika Głuch-Lutwin,[a] Tadeusz Karcz, Alicja Gawalska,[a] Michał Sapa,[a] Katarzyna Szafrańska,[a] Bartosz Pomierny,[a] Karolina Pytka,[a] Magdalena Kotańska,[a] Kamil Mika,[a] Marcin Kolaczkowski [a,b]

[a]Jagiellonian University Medical College, Faculty of Pharmacy, 9 Medyczna St., 30-688 Krakow, Poland

[b]Adamed Pharma S.A., Pienkow, 6A Mariana Adamkiewicza St., 05-152, Czosnow, Poland

*Corresponding Author Information:
Phone: (+48)126205460
E-mail: monika.marcinkowska@uj.edu.pl

1. NMR and UPLC-UV-MS spectra of selected compounds

2-(4-(3-(benzyloxy)-2-methylphenyl)piperazin-1-yl)ethan-1-ol (6)

^{1}H NMR (500 MHz, CHLOROFORM-d)

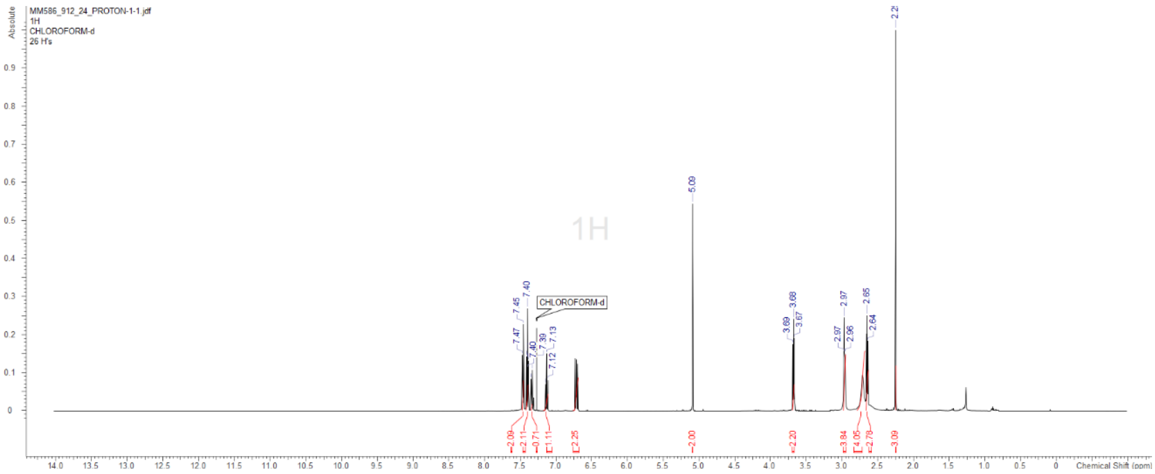

^{13}C NMR (126 MHz, CHLOROFORM-d)

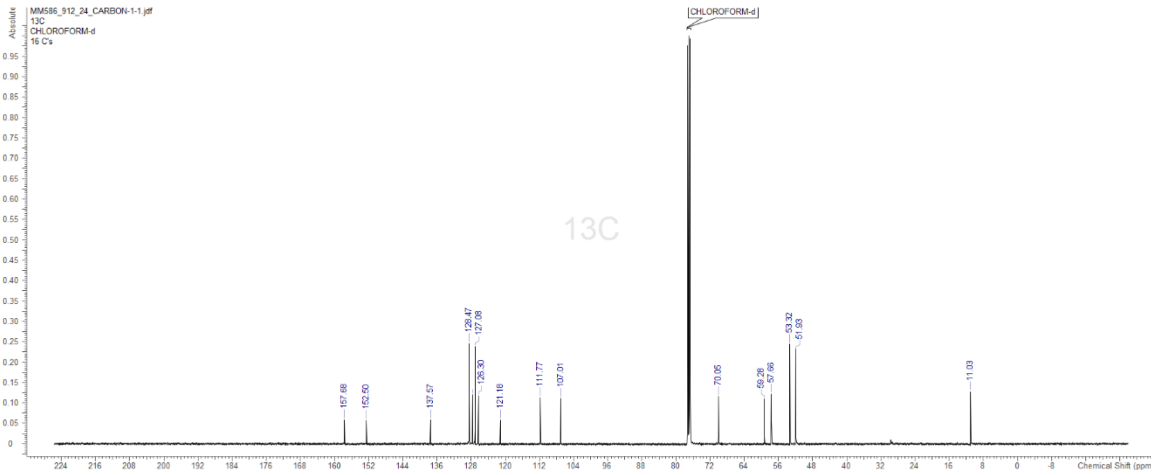

1-(2-((4-ammoniobutanoyl)oxy)ethyl)-4-(3-(benzyloxy)-2-methylphenyl)piperazin-1-ium dichloride (16)

^{1}H NMR (300 MHz, DMSO-d$_6$)

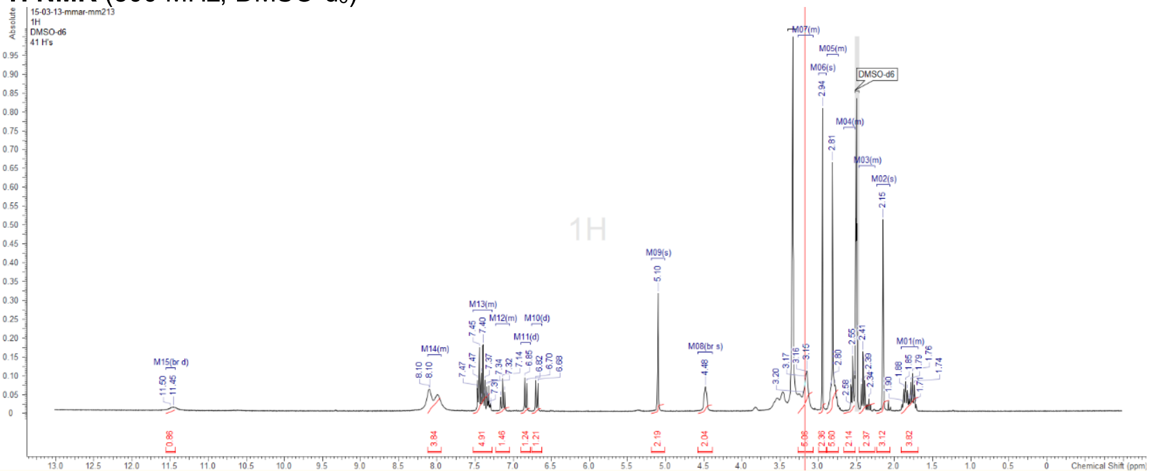

^{13}C NMR (75 MHz, DMSO-d$_6$)

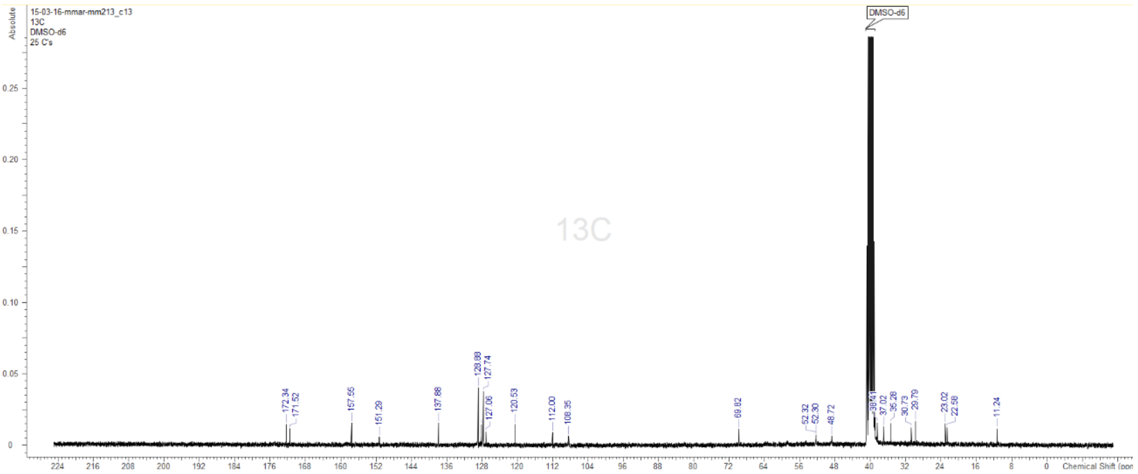

2. Predicted binding modes of selected molecules

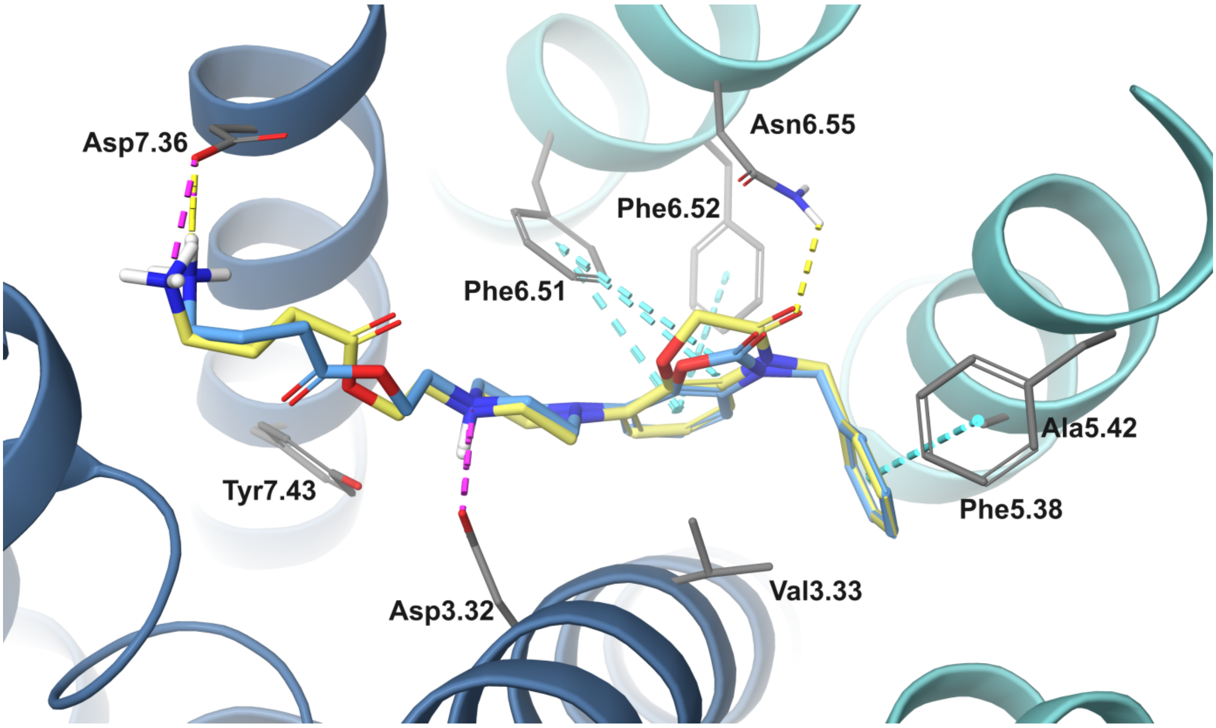

Figure S1. The predicted binding mode of hybrids **18** (blue) and **19** (yellow) at the binding site of 5-HT_6 receptor. Both ligands were aligned and formed crucial interactions – the ionic bond with Asp3.32 and π-π stackings with Phe6.51/Phe6.52 residues. The benzyl moieties formed π-π stackings with Phe5.38 in a hydrophobic pocket between Val3.33 and Ala5.42 residues. The extra carbon atom in the heterocyclic fragment of **19** changed the arrangement of the carbonyl group, leading to the formation of an important hydrogen bond with Asn6.55 side chain. This is believed to result in a 5-fold higher affinity of hybrid **19** (K_i = 25.0 ± 1.0) compared to **18** (K_i = 130.0 ± 12.0 nM)**.** GABA-fragments occupied the site between TMH1, TMH2 and TMH7, forming ionic interactions with Asp7.36 by protonated amine groups.

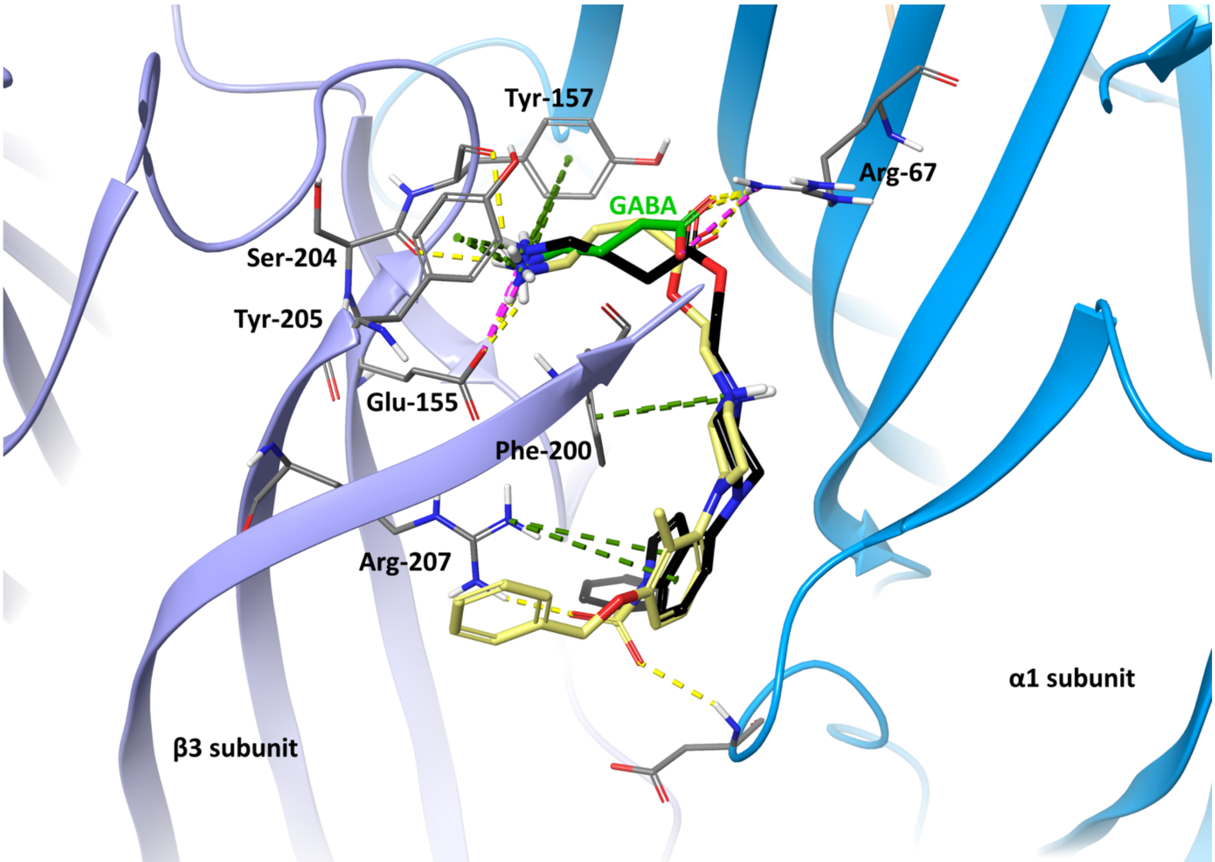

Figure S2. The predicted binding modes of hybrid **16** at the active site of the GABA-A receptor in comparison to the hybrid **3B**, the 1-(phenylsulfonyl)-4-(piperazin-1-yl)-1H-indazole derivative with high 5-HT6 receptor and GABA-A receptor agonistic activity (light yellow–16; green–GABA; black – 3B). The acyl fragments of both hybrids adopted a similar binding pose to the natural agonist in the GABA-A receptor and their analogous protonated piperazine rings formed cation-π interactions with β3Phe-200. The 1-(phenylsulfonyl)-1H-indazole moiety of hybrid **3B** created additional interactions with possibly significant β3Arg-207 residue: hydrogen bond interactions (sulfonyl group) with β3Arg-207 and cation-π interactions, as well as α1Asp-184 (hydrogen bond with sulfonyl group). However, in the case of hybrid 16, only the cation-π interaction with β3Arg-207 (2-methylphenyl ring) was preserved.

3. Hepatotoxiticy assay

Table 1. The viability of cells

CONCENTRATION	100 µM		50 µM		10 µM		1 µM		0,1 µM	
COMPOUND	MEAN	CV	MEAN	CV	MEAN	CV	MEAN	CV	MEAN	CV
16	14%	2%	102%	0%	104%	0%	102%	0%	100%	0%

MEAN – The value are expressed as percentage of control (live cells).
CV- % Coefficient of variation
